# Image classification and reconstruction from low-density EEG

**DOI:** 10.1038/s41598-024-66228-1

**Published:** 2024-07-16

**Authors:** Sven Guenther, Nataliya Kosmyna, Pattie Maes

**Affiliations:** 1 School of Computation, Information and Technology, Technical University of Munich, Munich, Germany; 2 Media Lab, Massachusetts Institute of Technology, Cambridge, USA

**Keywords:** Computer science, Neural decoding

## Abstract

Recent advances in visual decoding have enabled the classification and reconstruction of perceived images from the brain. However, previous approaches have predominantly relied on stationary, costly equipment like fMRI or high-density EEG, limiting the real-world availability and applicability of such projects. Additionally, several EEG-based paradigms have utilized artifactual, rather than stimulus-related information yielding flawed classification and reconstruction results. Our goal was to reduce the cost of the decoding paradigm, while increasing its flexibility. Therefore, we investigated whether the classification of an image category and the reconstruction of the image itself is possible from the visually evoked brain activity measured by a portable, 8-channel EEG. To compensate for the low electrode count and to avoid flawed predictions, we designed a theory-guided EEG setup and created a new experiment to obtain a dataset from 9 subjects. We compared five contemporary classification models with our setup reaching an average accuracy of 34.4% for 20 image classes on hold-out test recordings. For the reconstruction, the top-performing model was used as an EEG-encoder which was combined with a pretrained latent diffusion model via double-conditioning. After fine-tuning, we reconstructed images from the test set with a 1000 trial 50-class top-1 accuracy of 35.3%. While not reaching the same performance as MRI-based paradigms on unseen stimuli, our approach greatly improved the affordability and mobility of the visual decoding technology.

## Introduction

The past two decades have seen significant advances of classifying the object category of a perceived image and reconstructing visual stimuli from brain recordings^1–4^. The motivation for this has been two-fold. On the one hand, researchers hope to derive new insights into how the brain processes visual stimuli^5^. On the other hand, reconstructing visual information from someone’s brain could offer an intuitive communication channel for patients suffering from paralysis^6^. However, while classification accuracies and reconstruction qualities have steadily increased, two key challenges have remained largely unaddressed. First, previous studies have predominantly relied on costly and stationary equipment for functional Magnetic Resonance Imaging (fMRI), Magnetoencephalography (MEG) or high-channel Electroencephalography (EEG). While the monetary constraints limit the widespread use of these technologies, the immobility and long setup times of the equipment render a possible real-world application unfeasible. Second, most of the high-channel EEG studies have used a public dataset that presented all images of a class sequentially in blocks^7^. This allowed artifactual classifications based on block-level temporal correlations in the EEG signal rather than stimulus-associated patterns generated by the brain, as pointed out by Li et al.^8^. Thus, the models mostly learned to distinguish images not from the evoked signal, but from the temporal dynamics of the measurement tool.

Portable EEG devices may increase mobility and could greatly reduce the cost and preparation time with a reduction in electrode numbers. Arguably, a lower channel count corresponds to less information available for the classification. However, this effect may be mitigated by focusing on the most predictive electrode locations detected by previous research^9,10^. Additionally, the artifactual classifications of prior EEG studies could be avoided by randomly shuffling the stimuli after every experiment run during the data acquisition. Therefore, we propose to address the key challenges in the following three ways:

1. Reducing costs: To loosen financial constraints and enable more researchers to work with our findings, we employ a low-cost and commercially available EEG system.
2. Increasing flexibility: To avoid stationary devices or equipment with prolonged setup times, we utilize a portable, 8-channel EEG with a preparation time of less than 15 minutes. Furthermore, we use previous scientific findings to arrange the electrode positions in a theory-guided fashion for optimal use of the reduced channels. Thereby, we want to pave the way to the real-world usability of such systems.
3. Creating a new Image-EEG dataset: We run a new experiment with 9 subjects recording the EEG activity evoked by natural image stimuli to evaluate classification and reconstruction performances. We ensure the classification based on block-level temporal correlations is impossible by randomly shuffling the order of the presented images for every recording session.

Our study aims to implement the solutions mentioned above and to assess the classification and reconstruction performances using the new setup. Therefore, we first design the experimental paradigm to collect EEG-image pairs from 9 subjects. Subsequently, we adapt five current state-of-the-art EEG classification models to work with the streamlined recording system. We compare the different models to explore the boundaries of image object classification from EEG with our setup given the new dataset. Additionally, the classification accuracy is used as a validity measure to check how well our setup may discern the visually evoked potentials (VEP) of different image classes. Lastly, we attempt the reconstruction of the visual input stimuli from the EEG data. To achieve this, we modify the top-performing classifier to work as an EEG-encoder used to double-condition a pre-trained latent diffusion model (LDM) via additional projectors. We jointly finetune the EEG encoder, the projectors, and the cross-attention heads of the stable diffusion model, similar to Chen et al.^1^ who used embeddings extracted from fMRI recordings. Finally, we aim to answer whether the classification of an image category and reconstruction of the image itself is feasible from the visually evoked brain activity measured with a portable, low-density EEG.

## Methods

### Experiment

#### Subjects

Nine healthy participants (5 female), aged 20–33 (M: 22.5, SD: 1.8) were recruited at the research laboratory at Massachusetts Institute of Technology (MIT). All subjects were either students, research assistants, or research scientists at MIT. One subject was excluded for missing several recording sessions. All had normal or corrected-to-normal vision. Subjects received $100 compensation in two gift cards: $50 for participation and an additional $50 as a bonus, earned by correctly answering questions about presented images to increase motivation during the experiment. Informed consent from all subjects - for both study participation and publication of images during the study in an online open-access publication was obtained. The experimental protocol was approved by the Institutional Review Board of MIT.

#### EEG setup

**Figure 1:**
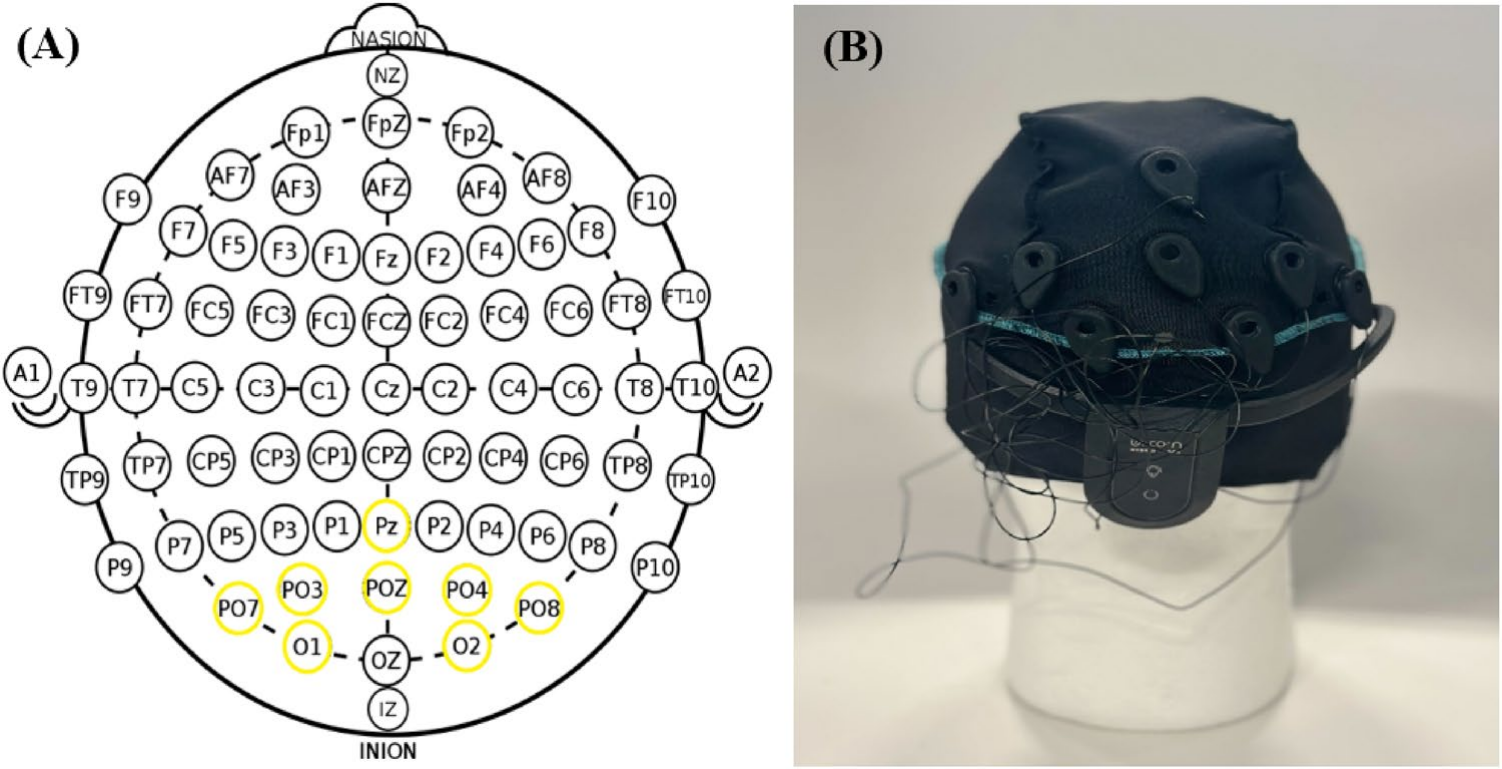
(**A**) 10–20 international system with the employed electrode locations highlighted in yellow (**B**) g.tec Unicorn Hybrid Black system configuration mounted onto an artificial head.

To record brain signals, we used the portable 8-channel g.tec Unicorn Hybrid Black EEG sampling at 250 Hz with an integrated amplifier. The electrodes of the Unicorn allowed for both dry and wet (gel-based) recordings. According to the most predictive channel locations identified in prior research^9,10^, we adapted the default channel positions of the Unicorn to allow an electrode positioning corresponding to the PO8, O2, O1, PO7, PO3, POZ, PO4, and Pz locations defined by the 10–20 international system^11^, respectively (Fig. 1A). The reference electrodes were placed on the left and right mastoids. The designed setup is displayed in Fig. 1B. We used the Unicorn Suite Hybrid Black software to ensure good signal quality and to visually inspect the signal. To collect the data stream from the EEG headset, we employed the Lab Streaming Layer (LSL)^12^.

#### Image selection

In our experiment, we presented 600 images, belonging to 20 image classes (30 per class) to the subjects during a session. 19 classes were a subset selected from the ImageNet Large Scale Visual Recognition Challenge^13^ and 30 human face images were added from a Kaggle dataset^14^ to create an additional Face category. This additional image category aimed to leverage the distinctive response human faces evoke in the visual cortex^9,15^. We ensured that the individual images per class only showed the respective object category and contained coherent low-level characteristics, like distinct silhouette and luminance properties, which have been shown to improve differentiation of classes^9^. The low-level coherence may be observed from the image class means shown in Supplementary Fig. 1. To facilitate the selection, we chose one prototypical image per class and selected 29 out of the 50 most similar images for the dataset. For details on the similarity score, see the Supplementary Materials.

#### Experiment setup

During the experiment, the 600 images were sequentially displayed for 2s each, separated by a 1s uniform gray screen to flush the visual percept of the preceding image. The way the brain encodes varying degrees of category abstraction unfolds sequentially over time^16^. Low-level information about displayed objects, like their shape, may be decoded as soon as 60ms post-presentation^17^, whereas high-level features, such as the category of the object, seem to be extractable only after 100ms^18^. Prior studies have used image exposure durations between 50ms^19^ and 2s^20^. We opted for the longer duration of 2s to reduce the risk of missing class discriminative information. Consequently, for the entire set of 600 stimuli, the session duration extended to 30 minutes. We selected the exposure time and number of images to strike a balance with regards to the session duration, ensuring that the experiment remained sufficiently brief for participants to sustain their attention. Analogous to the most frequently used dataset^7^ for visual decoding, we decided to keep the number of samples per class higher than the number of classes. However, in contrast to this dataset, we prevented classifications based on block-level temporal correlations by randomly shuffling the image presentation order for each session. To run the experiment, we used the Psychopy software^21^ and utilized the LSL LabRecorder to combine and synchronize the experiment and EEG streams. All processing steps and analyses were conducted using Python.

**Figure 2:**
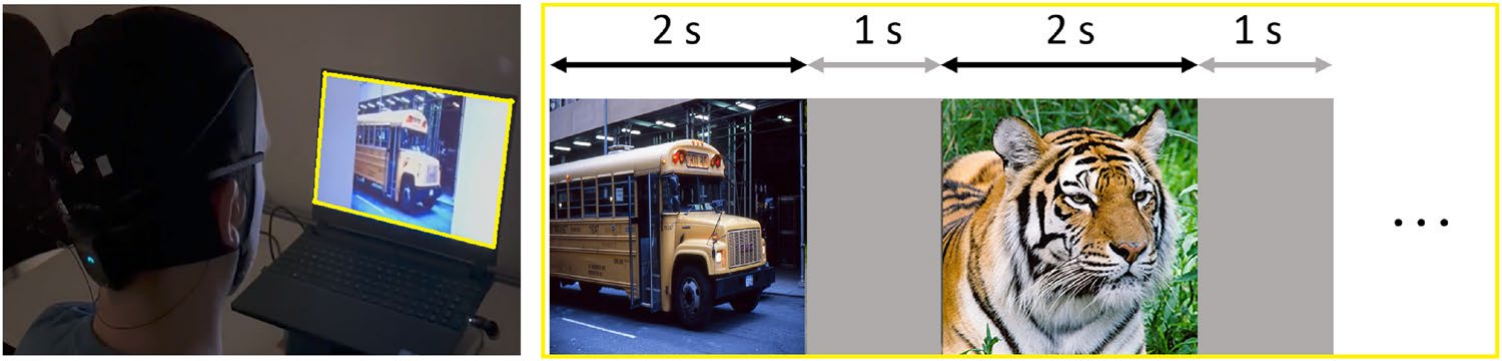
Subject sitting in front of experiment laptop with images being presented for 2s each with 1s gray screen in between.

The experiments were conducted in a darkened experiment room with no windows at the MIT Media Lab to minimize external visual distractions while subjects focused on the screen. After finishing the EEG setup, the participant took a comfortable seating position with the 15.6” experiment screen on a desk in front of them at a distance of roughly 0,5 m. Subjects were instructed to sit still and the screen was adjusted such that the top matched the eye level of the participant. After the setup, the experimenter left the room upon which the subject could start the experiment by clicking the space bar on a keyboard in front of them by themselves. The experimenter was not present during the recordings. Usually, between one to three sessions were recorded in a row, depending on how attentive the subject felt (self-reporting). In between sessions, we checked the signal quality using the Unicorn Recorder. Each subject underwent a total of 12 sessions of wet recordings. For the first subject, we additionally analyzed 12 dry recordings to evaluate the necessity of using conductive gel in the experiment. Fig. 2 displays the experiment process.

### Preprocessing

After collecting the EEG data from each subject, we applied a preprocessing pipeline to increase the signal-to-noise ratio (SNR) of the recordings and to create a data format suitable for the subsequent classification. The pipeline was applied to each recording session separately and consisted of the rejection of bad trials, filtering, channel-wise z-normalization, and clamping (>20 std. dev.). A trial was regarded as the EEG activity accompanying an image from its onset until it disappeared. Since each image was presented for two seconds, we selected the 500 EEG samples after the image onset time to represent the trial. Therefore, each trial had a dimension of (8500), given the eight-channel setup of the EEG. The bad trial rejection was adapted from Bigdely-Shamlo et al.^22^ and involved the detection of trials with NaN values, flat signals or either too low or high inter-channel correlations. Its output was a mask marking bad trials. For the filtering we used a separate highpass (1 Hz), lowpass (95 Hz) and notch filter (60 Hz) applied before the trial segmentation to avoid edge artifacts^23^. Then, we segmented the data into trials again and used the previously calculated mask to exclude bad trials. Supplementary Table 1 shows the percentage of dropped trials, as well as the total number of trials per participant. For subjects 3, 4, and 7 one recording needed to be excluded due to a malfunctioning electrode. Lastly, we inspected the preprocessed data to visually verify that it contained typical VEPs (Supplementary Fig. 3). A detailed description of the bad trial rejection and filtering is given in the Supplementary Materials.

### Classification

Following the preprocessing of the EEG data to ensure signal quality, we aimed to classify the image class of the perceived images from the associated brain activity. To this end, we compared five state-of-the-art EEG classification models: (1) EEGNet^24^, (2) TSCeption^25^, (3) EEG-ChannelNet^26^, (4) EEG Conformer^27^, and (5) the EEG-to-image transformation approach by Mishra et al.^28^. For ease of reference, we will refer to all but the EEG-to-image classification model as ‘deep classifiers’, as they use a neural network to arrive at the final classification. While the deep classifiers all utilize convolutional neural networks (CNN) for the classification, their architectures differ at fundamental levels. The EEGNet is a compact CNN that uses 2D convolutions over the time dimension, as well as depthwise convolutions along the spatial dimension to obtain frequency-specific spatial filters followed by a separable convolution layer^29^ to extract complex feature maps from the EEG signal. It has been validated on multiple EEG-based paradigms, including various VEP classification studies^4,20^. In turn, TSCeption draws inspiration from GoogleNet’s inception block^30^, employing multiple temporal and spatial convolutional kernels, chosen based on sampling-rate ratios and channel-locations, respectively, for diverse feature learning. EEG-ChannelNet combines elements from EEGNet and TSCeption and was conceptualized to improve upon the EEGNet in visual classification. It contains multiple temporal and spatial filters, like TSCeption, but utilizes different dilated kernels to capture various temporal patterns. Most notably, the EEG-ChannelNet aims to extract complex spatio-temporal representations by adding a block of residual layers consisting of 2D convolutions after the initial temporal and spatial filtering. The EEG Conformer mainly differs from the other models by introducing self-attention modules after its CNN backbone to improve the detection of global patterns in the signal. Finally, the EEG-to-image-based model stands apart by transforming EEG recordings into grayscale images, leveraging a pre-trained image classification model for feature extraction and using a machine learning classifier as output.

Except for the EEGNet and the EEG-to-image classification algorithms, we obtained the original implementations and adapted them to work in our framework. For the EEGNet, we employed the implementation from Braindecode^31^. The EEG-to-image transformation strategy was constructed according to the description given by Mishra et al.^28^ and Zhang et al.^32^. The subject-wise classification models were trained on all but two recordings per subject which served as hold-out validation and test set, respectively. The objective during training was to minimize the cross-entropy loss for which we used the Gradient Descent algorithm including Adaptive Movement Estimation (Adam)^33^ and Weight Decay (L2 penalty)^34^. Additionally, we employed a one-cycle learning rate scheduling^35^ for faster convergence, limiting the number of training epochs to 30 for all deep classifiers, except for the EEG Conformer. Notably, we investigated different hyperparameter combinations for each model, as explained in the Supplementary Materials. All models were trained on a NVIDIA GeForce RTX 3070 GPU.

For each classification model and subject, we retrained the hyperparameter configuration yielding the best validation accuracy on the training and validation set. The resulting model was used once on the test set to obtain the final test result. Notably, in the data split, we selected the recording with the fewest missing trials as the test recording to accurately estimate accuracies for each image class. To estimate the selection effect, we conducted a k-fold cross-validation run for every subject, where k equaled the number of non-test recordings. During this approach, the best-performing model was iteratively fitted on k-1 recordings and evaluated on the left-out recording before averaging across the accuracies. We then used a Wilcoxon Signed-Rank Test to examine whether the accuracy on the selected test recordings was statistically significantly higher than the average accuracy on the cross-validation runs. Additionally, we were looking for the best classification algorithm which was supposed to be adapted to work as an EEG encoder for the reconstruction task. Therefore, we examined whether the best model obtained a significantly higher accuracy than the other models with a Wilcoxon Signed-Rank test. We corrected for multiple testing using Bonferroni, yielding a Type 1 error probability of $\alpha =0.01$. We opted for the non-parametric test for its robustness against violations of the normality assumption, which was especially relevant given the small sample size of n = 8.

### Reconstruction

#### Latent diffusion model

To generate images, we employ a latent diffusion model (LDM)^36^. A Diffusion Model (DM)^37^ is a probabilistic model consisting of a forward and backward process. For image generation, the forward process incrementally adds noise to an image over a series of steps until the input is turned into Gaussian noise. In the backward process, the model aims to gradually remove the noise in a step-wise fashion until a sample in the original input data distribution is received. The denoising can be described as the reverse process of a Markov Chain of T steps, where the states, $t=1,..., T$, represent progressive additions of noise in the forward process. At each step in the backward process, a denoising function, $\epsilon _\theta (x_t,t)$, takes in a noisy version, $x_t$, of the input, *x*, and predicts a denoised version, $x_{(t-1)}$. The denoising function is commonly realized as a UNet^38^. Because operating in pixel space comes with a high memory demand, Rombach et al.^36^ have suggested to feed the input image through a Vector Quantized-Variational Autoencoder (VQ-VAE)^39^ to obtain a lower dimensional representation. Namely, the encoder, ${\mathcal {E}}$, of the VQ-VAE is used to reduce the dimensionality of the high-dimensional image, $x \in {\mathbb {R}}^{HxWx3}$, to its latent representation, $z={\mathcal {E}}(x)$. Subsequently, *z* is passed to the DM and its (latent) output is decoded back to the image space by the VQ-VAE’s decoder, ${\mathcal {D}}$.

Additionally, Rombach et al.^36^ have introduced a conditioning mechanism into the diffusion process to control the generation of images from other inputs, *y*, like text. This is realized by employing a domain-specific projector, $\tau _\theta$, such that $\tau _\theta (y) \in {\mathbb {R}}^{Mxd_\tau }$ can be linked to cross attention layers in the modified UNet, $\epsilon _\theta (z_t,t,\tau _\theta (y))$. M is an adjustable parameter. The attention is modeled as

1 $$\begin{aligned} Attention(Q,K,V)=Softmax(\frac{QK^T}{\sqrt{d_k}})\cdot V \end{aligned}$$

where $Q=W_Q^{(i)}\cdot \varphi _i(z_t)$, $K=W_K^{(i)}\cdot \tau _\theta (y)$, $V=W_V^{(i)}\cdot \tau _\theta (y)$ and $W_Q^{(i)} \in {\mathbb {R}}^{dxd_\tau }$, $W_K^{(i)} \in {\mathbb {R}}^{dxd_\tau }$, $W_V^{(i)} \in {\mathbb {R}}^{dxd_\epsilon ^i}$ are trainable projection matrices. Q, K, and V are also known as query, key, and value, respectively. $\varphi _i(z_t)$ marks an intermediate layer of $\epsilon _\theta$. For an *x*-*y* input pair, the conditional LDM can be trained with the objective

2 $$\begin{aligned} L_{LDM}:={\mathbb {E}}_{{\mathcal {E}}(x),y,\epsilon \sim {\mathcal {N}}(0,I),t}[\Vert \epsilon -\epsilon _\theta (z_t,t,\tau _\theta (y))\Vert _2^2] \end{aligned}$$

#### Reconstructing images from the brain via double-conditioned LDM

For the reconstruction of the perceived images from the evoked brain signals, we adapted the framework by Chen et al.^1^, which utilized fMRI representations to double-condition a pretrained LDM. In our case, the domain-specific projector, $\tau _\theta$, takes an EEG embedding as input and feeds to the cross-attention heads in the UNet. Additionally, another projector, $\sigma _\theta$, is used to obtain $\sigma _\theta (\tau _\theta (y))\in {\mathbb {R}}^{1xd_t}$, matching the time embedding dimension, $d_t$. Thereby, the EEG embedding may be added to the time step embeddings in the UNet for additional time steps conditioning^40^. Thus, the optimization objective becomes

3 $$\begin{aligned} L_{LDM}:={\mathbb {E}}_{{\mathcal {E}}(x),y,\epsilon \sim {\mathcal {N}}(0,I),t}[\Vert \epsilon -\epsilon _\theta (z_t,t,\tau _\theta (y),\sigma _\theta (\tau _\theta (y)))\Vert _2^2] \end{aligned}$$

#### EEG encoder

**Figure 3:**
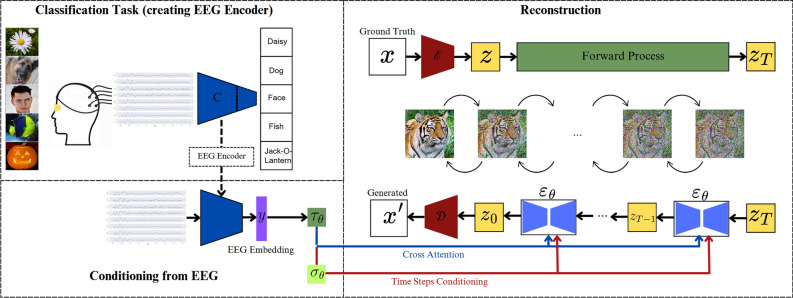
In the classification task, we aimed to predict the image class from VEPs. The best performing classifier, C, was adapted to work as an EEG encoder which we used to condition the pretrained LDM with the resulting EEG embedding, *y*. Therefore, we employed two projectors, $\tau _\theta$ and $\sigma _\theta$, which respectively matched the dimensions expected by the cross-attention heads and the time embeddings in the UNet, $\epsilon _\theta$, for cross-attention and time steps conditioning. The iteratively (de)noised images shown in the reconstruction were only used for illustrative purposes, as the actual LDM works in the latent image space.

To obtain an EEG encoder, we modified the best-performing model by exchanging its classifier head with two linear layers. The first layer contained 512 nodes and was used as an embedding, while the second layer was utilized as the classifier output. To ensure this adapted model maintained comparable classification accuracy, we repeated the hyperparameter optimization with the same data split as used for the prior models and evaluated the model performance on the test set for each subject. The data and model of the subject with the best accuracy were then selected for reconstruction. We employed the best performing model anticipating it would most effectively extract class discriminative information, while expecting its efficiency to be a limiting factor to the reconstruction performance. We removed the final classification layer and used the rest of the model as the EEG encoder. Whereas Chen et al.^1^ employed an embedding dimension of 1024 which was halved in the linear projector to match the dimensions of the LDM, we reduced the computational complexity by directly mapping to a dimension of 512 in the EEG encoder and using a 1x1 convolution projector to match the expected depth. Fig. 3 presents the different stages employed for the reconstruction, from learning the EEG encoder to conditioning the pretrained LDM for the image generation.

#### Finetuning

The LDM was pre-trained in a separate conditioning context, therefore, we had to finetune it to use the encoded EEG signals to condition the generative model. To fine-tune the pre-trained LDM, we employed the EEG-image pairs of the training and validation set of the subject for training and validation, respectively. However, we only updated the EEG encoder, as well as the cross-attention and projection heads, similar to Chen et al.^1^. Thus, for a given EEG-image pair, the VQ-VAE encoder turned the image into the latent space representation, which was then employed as the objective during training. The associated EEG signal was transformed into an embedding and passed to the cross-attention modules via the convolution projector, where it functioned as the key and value. Additionally, the projected embedding was added to the time embedding in the UNet for time-step conditioning. For the training, we adopted the procedure and hyperparameters from Chen et al.^1^, but employed a smaller learning rate of 5e-6 and only fine-tuned for 200 epochs. Similar to the classification task, the final fine-tuned model was trained on the train and validation set and evaluated on the test recording. The finetuning was able to run on a NVIDIA GeForce RTX 3070 GPU.

#### Image generation and evaluation

Eventually, the fine-tuned model could be used to reconstruct perceived images from associated EEG signals. The images were generated in a 256x256 pixel format using 250 sampling steps with the Pseudo Linear Multi-Step (PLMS) method^41^. For each EEG-image pair in the test set, we generated five reconstructions with different random states and reported the 1000 trial 50-class top-1 accuracy on the best-generated sample per image, as in Chen et al.^1^. Additionally, we calculated the mean 50-class top-1 accuracy across the five samples per image to evaluate the generation consistency. The utilized image classifier in the metric was a vision transformer^42^.

However, as the image classes in the test set were the same used in the training and validation sets, this approach did not allow a direct comparison to the reconstructions done by Chen et al.^1^. Therefore, we obtained an additional recording of the best-performing subject but presented 300 images from 10 object categories (30 images per class) that were not used during training. This yielded another test recording with unseen image classes to test the model’s ability of zero-shot learning. To clarify which test set we refer to in the remainder of this study, we will call the test set that shared image classes with the training and validation set the “base test set”. The other test recording will be coined in this paper as the “advanced test set”. Similar to the base test set, we prepared the advanced test set with the preprocessing pipeline. We report the results on both test sets to estimate the capability of our approach to reconstruct images from known, as well as unknown image categories.

Additionally, we further aimed to investigate the effect of the classification model on the reconstruction outcome. First, we tested our expectation that the performance of the classification model which we used for the EEG encoder would limit the reconstruction performance. Therefore, we computed the point-biserial correlation between the classifier performance per image (0 for misclassification; 1 for correct classification) and the reconstruction performance as evidenced by the 1000 trial 50-class top-1 accuracy on the best-generated sample per image. Second, we examined whether the reconstruction depended on the actual EEG input, instead of being driven solely by the model. For this we used the inter-trial EEG recordings, which contained no stimulus-associated signal, as input for the reconstruction. Since the inter-trial durations were only 1s long, we upsampled them to obtain the same length as for the original input and applied the channel-wise normalization.

The code to replicate the data acquisition, preprocessing, classification, and reconstruction conducted in this study is openly accessible via the following link: https://github.com/mitmedialab/eegreconstruction.

### Institutional review board 

The study was conducted in accordance with the Declaration of Helsinki, and approved by the Institutional Review Board of MIT (protocol code 2107000428A003, 09/15/2021).

## Results

### Classification

**Figure 4:**
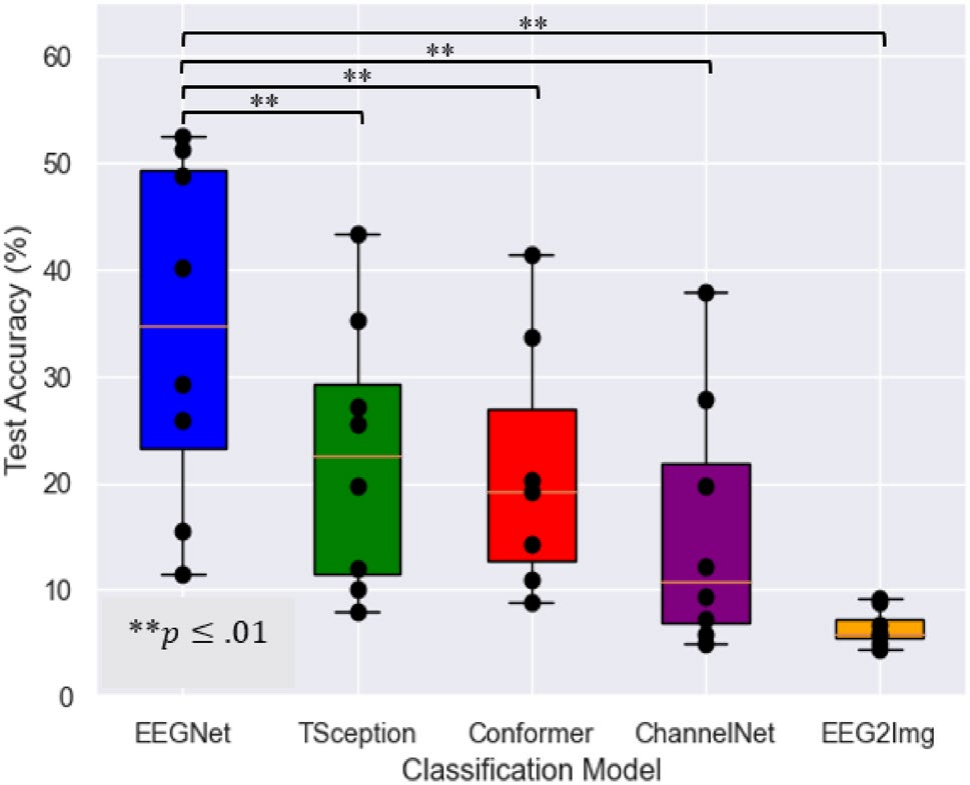
Test accuracy across subjects for the separate classification models.

With a test accuracy of 34.4% averaged across subjects, the EEGNet model significantly outperformed all other classifiers ($p<.01$). It was followed by the TSception and the EEG Conformer with a mean performance of 22.6% and 21.2%, respectively. In contrast, the EEG ChannelNet only reached 15.6% accuracy, while the EEG-to-Image paradigm ranged just around chance level with 6.4%. The comparison is shown in Fig. 4. To estimate the effect of our test set selection bias, we compared the mean average cross-validation result on the training and validation sets of each subject with the mean test set accuracy. Using the EEGNet, the average accuracy on the test sets (34.4%) was higher than that of the cross-validation (31.0%), however, the difference was not statistically significant ($p=0.054$).

Table 1 shows the best test accuracies for each model and subject.The performance differed strongly across participants with a standard deviation of 15.1% for the EEGNet. Notably, the EEGNet was not only the best performing model but also the most consistent one regarding the hyperparameter search. Namely, the most complex architecture possible in the search with 64 temporal filters, max pooling and a Dropout probability of 0.25 yielded the superior result across subjects. Furthermore, we compared the classification performance with the EEGNet on the wet and dry recordings of Subject 1. Whereas the test accuracy on the wet recordings reached 52.5%, the same model only achieved 24.2% on the dry measurements.

**Table 1:** Classification accuracy in percentage on hold-out test recording for each model and subject, respectively.

	P001	P002	P003	P004	P005	P006	P007	P008	Mean (SD)
EEGNet	**52.5**	**40.2**	**15.5**	**51.2**	**11.5**	**29.3**	**48.7**	**25.9**	**34.4 (15.1)**
TSCeption	43.3	25.5	12.0	35.3	8.0	19.7	27.2	10.1	22.6(11.8)
Conformer	41.3	19.3	11.0	33.7	8.8	20.2	29.8	14.3	22.3 (10.8)
ChannelNet	37.9	12.1	7.3	27.8	5.8	9.3	19.8	5.0	15.6(11.4)
EEG-to-Img	5.0	5.7	5.7	9.2	6.0	6.7	8.8	4.5	6.4(1.6)

Best result of each subject is highlighted in bold. The last column shows mean accuracy and standard deviation for each model.

With the best performance per subject highlighted in bold.

**Figure 5:**
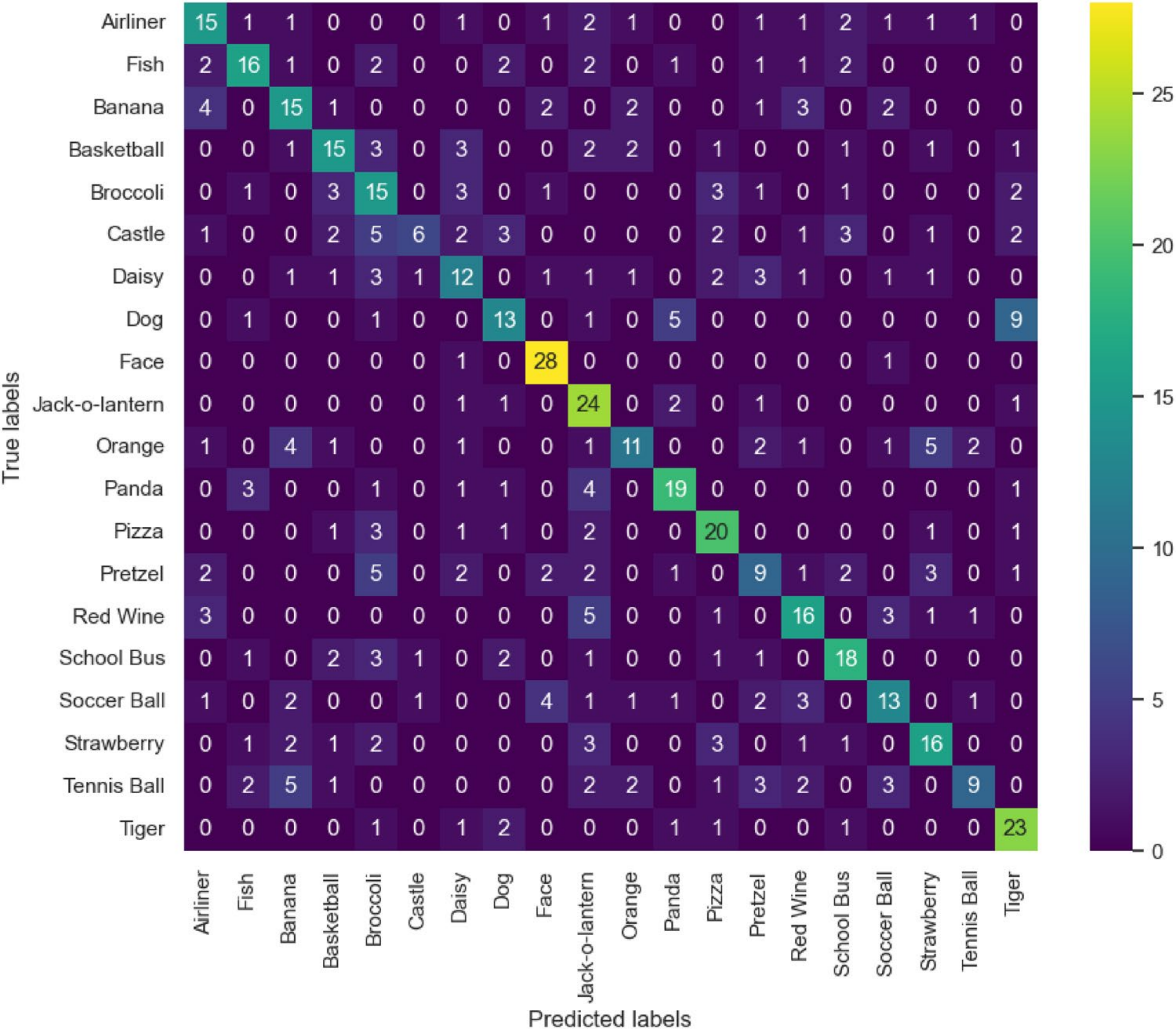
Confusion matrix displaying true and predicted labels for each image class for the EEGNet model on the test recording from Subject 1. Ground truth labels are presented on the y-axis and predicted labels on the x-axis.

Not surprisingly, the *Face* category obtained the highest accuracy (55.42%) of all image classes, as shown in Table 2. Across subjects, the *Face* class was followed by the *Red Wine* and *Airliner* image category. The least successfully predicted class was the *Pretzel* category with 20.86% accuracy. Additionally, we computed the confusion matrix for the best performing subject/model combination (Subject 1, EEGNet), presented in Fig. 5, to investigate common misclassifications. For subject 1, the image classes containing facial features (*Face* and *Jack-o-Lantern*), as well as the animal categories (*Panda* and *Tiger*) obtained the highest accuracies. In turn, the *Castle* class seemed to be hardest to classify. Common misclassifications occurred between semantically similar image categories, like the Dog class which was frequently misinterpreted as *Panda* or *Tiger*.

**Table 2:** Average classification accuracy (%) and standard deviation across subjects with EEGNet model for each image class.

Image class	Mean accuracy
Airliner	46.88 (29.09)
Fish	29.17(18.32)
Banana	23.75 (19.80)
Basketball	29.58 (17.31)
Broccoli	37.50 (23.08)
Castle	29.35 (17.61)
Daisy	27.85 (11.19)
Dog	28.33 (17.00)
Face	55.42 (28.95)
Jack-o-Lantern	35.00 (24.49)
Orange	28.33 (15.33)
Panda	29.17(22.24)
Pizza	46.67 (23.44)
Pretzel	20.86(12.52)
Red Wine	50.00 (23.23)
School Bus	41.25 (23.63)
Soccer Ball	22.14(17.05)
Strawberry	31.67 (15.53)
Tennis Ball	27.50(16.88)
Tiger	45.42 (21.52)

### Reconstruction

For the reconstruction task, we modified the best-performing model (EEGNet) to obtain the EEG encoder. For simplicity, we called the new model EEGNet+. To verify that EEGNet+ retained a similar performance as the initial EEGNet, we evaluated it on the base test set for each subject. With an average test accuracy of 34.6%, its performance did not differ significantly from EEGNet ($p=0.61$). After pretraining the EEGNet+ model in the classification task, we discarded the output linear layer to obtain our EEG encoder and combined it with the pre-trained LDM. Upon fine-tuning the EEG encoder, as well as the attention and projection heads, we tried to reconstruct the images from their associated EEG signals. We will now report the results on Subject 1, who had the best performance in the classification task. The reconstruction results for an additional subject (subject 7) can be found in Supplementary Fig.  13.

**Figure 6:**
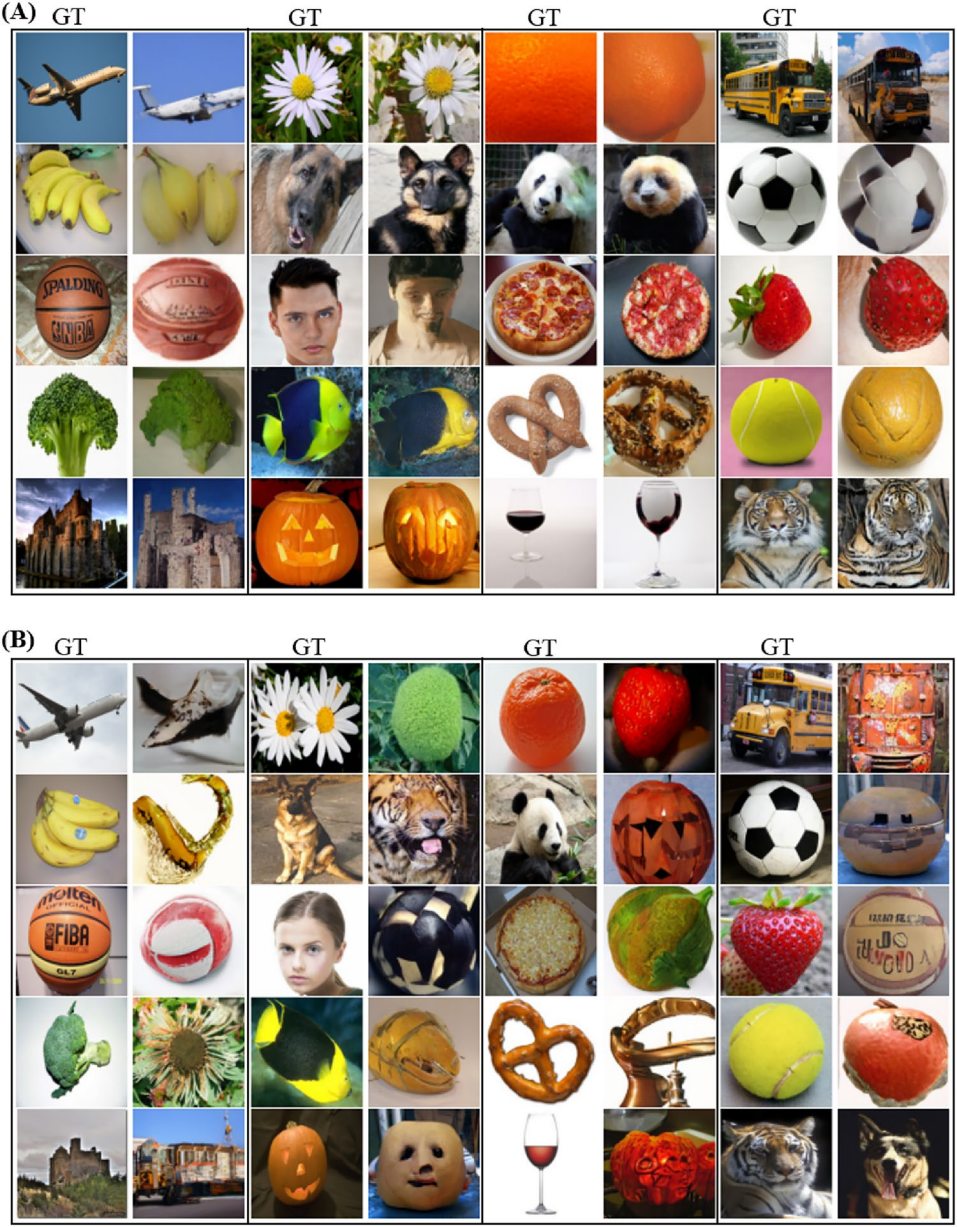
Selection of (**A**) good reconstructions and (**B**) typical failed reconstructions for an example of each image class from the base test set. Ground truth (GT) is presented on the left with the reconstructed image to the right for each column, respectively. The presented ground truth images, except for the human face image, were taken from the ImageNet Large Scale Visual Recognition Challenge^13^ dataset which permits usage for non-commercial research. The human face image was taken from the Human Faces dataset^14^ published under CC0 license.

On the base test set, the 1000 trial 50-class top-1 accuracy on the best-generated images, as well as the average 50-class top-1 accuracy across the five generated samples per image, were 35.3% and 30.9%, respectively. Fig. 6 displays a selection of good and bad reconstructions of our approach. Similar to the classification task, the reconstruction of the *Face* images was the best, even though facial details were often not represented correctly. Additionally, the reconstruction of the pictures showing an animal, especially for the *Tiger* and *Panda* classes, worked better than for most inanimate objects. Notably, in many cases during which the generated images differed from the ground truth, the shape, but not the color, seemed to be correct. Moreover, the incorrectly generated samples often depicted one of the other object categories in the dataset. In other cases, the falsely reconstructed image contained a mix of the ground truth and a different class, like a jack-o-lantern with a panda face. These phenomena can be observed in the reconstructions shown in Fig. 6B.

**Figure 7:**
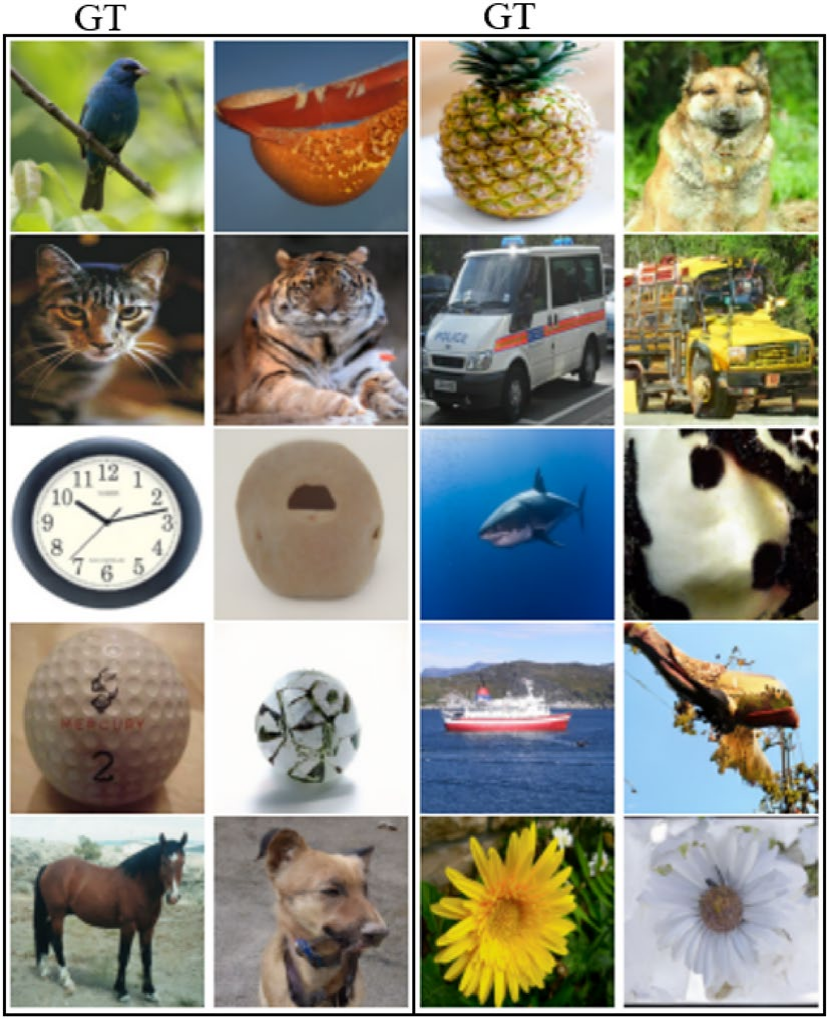
Selection of reconstructions for an example of each image class from the advanced test set. Ground truth is presented on the left with the reconstructed image to the right for each column, respectively. The presented ground truth images were taken from the ImageNet Large Scale Visual Recognition Challenge^13^ dataset which permits usage for non-commercial research.

The advanced test set contained previously unseen image categories (*Blue Bird*, *Cat*, *Clock*, *Golf Ball*, *Horse*, *Pineapple*, *Police Truck*, *Shark*, *Ship*, and *Sunflower*). On this test set, we achieved a lower 1000 trial 50-class top-1 accuracy of 8.2% on the best-generated images and an average of 7.2% across the five generated samples per image. For the advanced test set, the generated images were highly variable and usually depicted the wrong image class which had a similarity to the image categories that were used to finetune the LDM. Fig. 7 shows a selection of reconstructions for each image category in the advanced test set. Notably, for some of the new image classes that exhibited similarity to the categories employed during training (e.g., *Cat* and *Tiger*), the model consistently generated its corresponding class from the set of previously observed categories. While some of the reconstructions matched the shape of the ground truth image, this was not always the case, as can be seen for the *Blue Bird* or *Shark* image.

Furthermore, we tested whether the performance of the classifier utilized as EEG encoder would affect the reconstruction results. We found a correlation of $r=0.48$, $p<0.001$ between the ability of the classifier to correctly classify an image and the reconstruction performance for that image as measured by the 1000 trial 50-class top-1 accuracy on the best-generated sample. The reconstruction based on the inter-trial signals yielded a 1000 trial 50-class top-1 accuracy of 4.5% on the best-generated images and an average 1000 trial 50-class top-1 accuracy across the five generated samples per image of 3.2%.

## Discussion

The goal of our study was to investigate whether we could classify the category of a perceived image from its VEP and reconstruct the image, utilizing a portable, low-density EEG. Our findings, highlighted by the standout performance of the EEGNet model, demonstrate the feasibility of employing a low-density EEG system for discerning image classes from VEPs. Additionally, the successful generation of images from the base test set displays the capability of our simplified setup to reconstruct images from image classes that have been used during fine-tuning. Notably, for both classification and reconstruction, the outcomes were the best for image categories with facial features or those depicting animals. However, the reconstruction efforts were confined to image classes encountered during training, as evidenced by the drastically lower 1000 trial 50-class top-1 accuracies. Thus, while our setup shows promise for visual decoding of certain image classes, its effectiveness is limited if unseen classes are introduced during testing. It remains to be established whether this limitation is due to the use of a low-density EEG system, or pertains to other decisions taken in this study, as discussed below.

Regarding the key challenges, we believe to have greatly improved the cost and flexibility. Besides the affordable EEG hardware, the best classification model only contained about 62.000 parameters and even the reconstruction task could be fine-tuned on a single NVIDIA GeForce RTX 3070 Laptop GPU. Both the cost and efficiency increase the feasibility of replicating and extending our study by other labs and researchers. Additionally, we provided a new dataset that avoids the data acquisition mistakes made by prior studies^8^ and ascertained that the data contains stimulus-associated VEPs for which both the timing^9^^34^, as well as the typical N1-P2 onset response^43^ were in line with previous research. Similarly, the reduced accuracy for dry recordings was congruent with prior findings^44^.

A direct comparison with previous EEG-based VEP classification studies remains challenging. The most widely used EEG-image dataset^7^ has recorded the neural responses of 6 subjects observing 40 naturalistic image classes containing 50 samples per class with a 128-channel EEG. However, this dataset has given rise to several studies reporting extremely high accuracies of up to 99.5%^45^. As has been pointed out by several researchers^5,8^, these performances are a result of the utilized block design which enables to predict the image class not from the stimulus-evoked activity, but from the time-related changes in the measurement tool. Notably, this dataset has been employed in at least 10 studies^5^ and its use continues^46^. Thus, while our classification results were clearly above chance level prediction (5%), we struggle to put the performance in perspective with regards to absolute numbers. However, in relative terms, the studies that have utilized the EEG-ChannelNet^26^ and the EEG-to-Image paradigm^28^ on the aforementioned dataset have reported classification accuracies of 48% and 64%, respectively, which outperformed the EEGNet (32%). Interestingly, this pattern was reversed in our research with the EEGNet clearly outperforming the other two models and the EEG-to-Image approach ranging around chance prediction. While this could be partly due to the adaptations we made to the models, the phenomenon might be better explained by the artifactual predictions based on the block-level temporal correlations^8^. Namely, we had to make similar modifications with the EEGNet and explored several different model configurations for the EEG-ChannelNet and EEG-to-Image paradigm in the hyperparameter search. A more detailed discussion of why we think the EEG-to-Image framework was inferior can be found in the Supplementary Materials. To our knowledge our study was the first to use the TSCeption and the EEG Conformer model for visual decoding. Remarkably, both models exhibited similar classification performances despite their different architectures and outperformed the EEG-ChannelNet and EEG-to-Image framework. In general, one should note that the EEGNet model was a well-established CNN that has been tested in multiple EEG-based classification tasks, among which were several visual decoding studies^3,4^. In contrast, the other models have only been evaluated on the same flawed dataset (EEG-ChannelNet and EEG-to-Image) or were never used for visual decoding. Consequently, achieving optimal performance with these models might require more elaborate tweaking compared to the already optimized EEGNet.

As can be seen in Table 1, there was a high variability of classification performances between subjects. Unfortunately, inter-subjective variability is a common phenomenon in EEG studies^47^, but also in projects using other neuro-recording methods, like fMRI^1^. The potential reasons for this variability are multifaceted, including differences in age, sex, attention span, and anatomy, to name a few^48,49^. A different attempt to explain the inter-individual differences may be to look at the signal quality, as indicated by the relative number of bad trials that had to be dropped for a participant’s recordings. For this, we have plotted the classification accuracy against the percentage of dropped data and the total number of trials per person, respectively, in Supplementary Fig. 5. The Spearman rank-order correlation between the accuracy and percentage of dropped data and between the accuracy and the total number of trials were non-significant with $r=-0.24$, $p=0.57$ and $r=0.05$, $p=0.91$, respectively. Therefore, we do not see a connection between the signal quality, as indicated by the percentage of dropped data, and the classification performance in this study. However, these results should be treated with caution as the small sample size limits the potential for generalizations. Lastly, the subject’s motivation and attention during the experiment may play a vital role in the obtained results. While we tried to ensure optimal attention during recordings by minimizing distractions and offering incentives with the bonus compensation, we were not able to further account for this variable.

Regarding the classification accuracy per image class, it was not surprising that the *Face* category obtained the best results. As pointed out before, we specifically selected the *Face* image class as a stimulus category in this study as it was known to evoke distinct neural responses^9,15^. Surprisingly, across subjects, the *Red Wine* and *Airliner* categories had the best accuracy after the *Face* class. Previous studies have shown distinct brain responses to observing animate objects, compared to inanimate ones^50^. Furthermore, the property of looking like an animal has been shown to significantly explain unique variance in the EEG response to animate objects^51^. In theory, this would make the detection of the animal classes in our dataset easier, compared to images classes like *Red Wine*. In fact, this pattern was observed for subject 1 for which the *Tiger* and *Panda* classes performed better than most inanimate categories. Arguably, the *Dog* class was an exception, yet, a look at the confusion matrix clarifies that the majority of its misclassifications belonged to one of the other two animal categories. However, across participants, this effect was not observable. While the *Tiger* class obtained a high accuracy of 45.42%, the other animal classes ranged below 30% accuracy. A potential reason for the success rate of the *Red Wine* and *Airliner* categories might be the distinct background that these image classes had, as displayed in Supplementary Fig. 1. However, this assumption would require further testing. With regards to the *Pretzel* class which was hardest to classify, one should note the relatively high inter-class variability due to the different orientations of the *Pretzel* images. Possibly for this reason, the EEGNet model struggled to classify the associated VEPs correctly.

Concerning the reconstruction part of this study, we first evaluated the fine-tuned LDM on the base test set. As expected, the model worked best on images with facial features or animal classes, analogous to the classification task. However, the efficiency of the pretrained EEG encoder also limited the reconstruction which can be observed when one image class was mistaken as another one, like the generation of a tiger for the ground truth image of a dog in Fig. 6B. Additionally, we observed a positive moderate correlation between the classifier’s proficiency in accurately classifying an image and the 1000 trial 50-class top-1 accuracy calculated on the best reconstruction of the same image. This indicated that the reconstructions were superior for the images which the classifier predicted correctly. However, the embeddings did not only contain the predicted class information. The encoder clearly utilized other high-level information, like the shape and location of an object in the respective image. This becomes evident when inspecting the misclassifications in Figure 6B, where the rough shape and location of the ground truth were frequently preserved. In contrast, the color of the reconstructions was often wrong. A potential reason for this could be that the color of a perceived object seems to be encoded in the frontal rather than in occipito-parietal regions of the brain^52^. In turn, studies have shown that the object’s shape and texture are processed in V4^53^^54^, a part of the visual cortex that was located in the vicinity of our electrode locations. Notably, the mean 1000 trial 50-class top-1 accuracy across the five samples per image showed that the image reconstruction was consistent over sampling trials. Additionally, we showed that the reconstruction performance was not solely driven by the EEG encoder, but depended on the EEG input as indicated by the drastically lower accuracies for the inter-trial signals that were not associated with a presented stimulus.

To compare our approach to prior studies, we collected another test dataset with 10 previously unseen image classes. Compared to the 1000 trial 50-class top-1 accuracy of 27.4% achieved by^1^ and that of other fMRI-based reconstruction studies^55^, we were clearly below with 8.2%. Thus, our method was able to reconstruct images of classes observed during training, especially, images with facial features or the ones depicting animals. However, in contrast to the fMRI-based paradigms, we were not able to consistently reconstruct unseen image classes with our EEG setup. While there could be multiple reasons for that, the most obvious is the drastically lower spatial resolution of our EEG approach. While the temporal information of VEPs can be used to distinguish perceived image categories^18,56^, object processing is distributed across several brain areas^57,58^. Namely, there is a spatially discernable processing hierarchy in which early areas, like V1, encode low-level features, whereas higher layers of the visual cortex and structures in the inferotemporal cortex are responsible for the detection of complex shapes and object recognition^5^. Therefore, the poor spatial resolution of EEG and its limited ability to record from deeper brain structures inevitably misses additional information to correctly classify the perceived images. Moreover, the self-supervised paradigm employed in prior studies^1,55^ to pretrain the encoder might capture more general patterns that allow for better generalization to unseen image classes.

However, one should also take into consideration that most prior research involved costly equipment (fMRI) and would practically be impossible to work in real-world scenarios. Firstly, because of the bulky, stationary nature of the device, and secondly because of the hemodynamic response. With regards to the latter, one would need to wait multiple seconds before even observing the evoked activity in the fMRI signal. Additionally, the self-supervised techniques, like in Chen et al.^1^ employed much larger datasets (136000 fMRI segments) to pretrain the encoder on 8 RTX3090ti GPUs. While our approach is closer to practical applications due to its flexibility, affordability, and simplicity, there remain challenges to improving the proposed setup before transitioning it from a laboratory setting.

First, while we employed a portable and easy to setup device, we still used an artificial lab environment to minimize distractions. Future studies should explore the tradeoff between the increased flexibility and the reduction in the SNR, possibly allowing subjects greater freedom of movement during image presentation. Second, while improving on the temporal constraints of fMRI approaches, the real-time usage would still be limited for two reasons. Firstly, we employed a non-causal zero-phase filter in our preprocessing pipeline to preserve the signal’s temporal characteristics requiring future data to perform the filtering. Therefore, future studies should consider a causal filter when attempting the approach in real time. Secondly, the iterative inference process of the LDM required a significant computing time. Reducing the number of sampling steps might lessen the reconstruction quality and would still fall short of achieving real-time capability. However, there have been recent advances trying to enable real-time image generation using Adversarial Diffusion Distillation^59^, which could potentially address the aforementioned challenge. Third, our study solely focused on the feasibility of identifying and generating perceived images. However, for practical applications, the classification and reconstruction of ‘imagined’ (mentally visualized) images might be more relevant. A logical next step of our approach would be to test the pipeline with imagined rather than perceived images. As for this study, the image presentation and associated imagination should happen in a random order to avoid artifactual predictions. Fourth, our reconstruction performance was limited by the ability of the pretrained encoder to extract useful information to condition the LDM. While one possibility would be to look for superior classification models, this might not solve the problem. As has been shown in the fMRI-based reconstruction studies^1^^55^, an encoder that has been pre-trained in a self-supervised paradigm might pick up patterns that generalize better to brain signals evoked by unseen classes. Previously, Bai et al.^46^ employed masked signal modeling to pretrain an EEG encoder for reconstruction, however, the EEG-image data has been taken from the flawed block-design dataset^60^. Recently there have been further methods to create pretrained EEG models that could function as the encoder^61^.

While there remains a long road towards creating a fully practical end-to-end system that could reconstruct images from someone’s perception or visual imagination, we believe to have contributed a valuable step to bring such a system closer to the real world.

### Supplementary Information

Supplementary Information.

## Data Availability

The code of this study is openly available at https://github.com/mitmedialab/eegreconstruction. The data will be made available to everyone upon request via this form https://forms.gle/hXxuyGStUVgWrQbw9.

## References

[1] Chen, Z., Qing, J., Xiang, T., Yue, W. L. & Zhou, J. H. Seeing beyond the brain: Conditional diffusion model with sparse masked modeling for vision decoding. In 2023 IEEE/CVF Conference on Computer Vision and Pattern Recognition (CVPR), 22710–22720 (2022).

[2] Benchetrit, Y., Banville, H. & King, J.-R. Brain decoding: Toward real-time reconstruction of visual perception, 10.48550/arXiv.2310.19812 (2023). arXiv: 2310.19812.

[3] Lee S, Jang S, Jun SC (2022). Exploring the ability to classify visual perception and visual imagery eeg data: Toward an intuitive bci system. Electronics.

[4] Shimizu H, Srinivasan R (2022). Improving classification and reconstruction of imagined images from eeg signals. PLoS ONE.

[5] Holly Wilson, M. G. M. J. P., Xi Chen & O’Neill, E. Feasibility of decoding visual information from eeg. Brain-computer interfaces, 1–28, 10.1080/2326263X.2023.2287719 (2023).

[6] Van Den Boom MA, Vansteensel MJ, Koppeschaar MI, Raemaekers MAH, Ramsey NF (2019). Towards an intuitive communication-BCI: Decoding visually imagined characters from the early visual cortex using high-field fMRI. Biomed. Phys. Eng. Express.

[7] Spampinato, C. et al. Deep learning human mind for automated visual classification. In In 2017 IEEE conference on computer vision and pattern recognition (CVPR), 4503–4511, 10.1109/CVPR.2017.479 (2017).

[8] Li R (2021). The perils and pitfalls of block design for eeg classification experiments. IEEE Trans. Pattern Anal. Mach. Intell..

[9] Kaneshiro B, Perreau Guimaraes M, Kim H-S, Norcia AM, Suppes P (2015). A representational similarity analysis of the dynamics of object processing using single-trial eeg classification. PLOS ONE.

[10] Simanova I, van Gerven M, Oostenveld R, Hagoort P (2011). Identifying object categories from event-related eeg: Toward decoding of conceptual representations. PLoS ONE.

[11] Klem GH, Lüders H, Jasper HH, Elger CE (1999). The ten-twenty electrode system of the international federation the international federation of clinical neurophysiology. Electroencephal. Clin. Neurophysiol..

[12] Kothe, C. Lab streaming layer (lsl) - a software framework for synchronizing a large array of data collection and stimulation devices. Computer software (2014).

[13] Russakovsky O (2015). Imagenet large scale visual recognition challenge. Int. J. Comput. Vision.

[14] Gupta, A. Human faces [dataset]. Kaggle (2021 (Accessed January 10, 2024)). https://www.kaggle.com/datasets/ashwingupta3012/human-faces.

[15] Nichols D, Betts L, Wilson H (2010). Decoding of faces and face components in face-sensitive human visual cortex. Front. Psychol..

[16] Contini EW, Wardle SG, Carlson TA (2017). Decoding the time-course of object recognition in the human brain: From visual features to categorical decisions. Neuropsychologia.

[17] Teichmann L (2020). The influence of object-color knowledge on emerging object representations in the brain. J. Neurosci..

[18] Carlson T, Tovar DA, Alink A, Kriegeskorte N (2013). Representational dynamics of object vision: The first 1000 ms. J. Vision.

[19] Grootswagers T, Zhou I, Robinson AK, Hebart MN, Carlson TA (2022). Human EEG recordings for 1,854 concepts presented in rapid serial visual presentation streams. Sci. Data.

[20] Lee S, Jang S, Jun SC (2022). Exploring the ability to classify visual perception and visual imagery eeg data: Toward an intuitive bci system. Electronics.

[21] Peirce JW (2019). Psychopy2: Experiments in behavior made easy. Behav. Res. Methods.

[22] Bigdely-Shamlo N, Mullen T, Kothe C, Su K-M, Robbins KA (2015). The prep pipeline: Standardized preprocessing for large-scale eeg analysis. Front. Neuroinform..

[23] van Driel J, Olivers CN, Fahrenfort JJ (2021). High-pass filtering artifacts in multivariate classification of neural time series data. J. Neurosci. Methods.

[24] Lawhern VJ (2018). EEGNet: A compact convolutional neural network for EEG-based brain-computer interfaces. J. Neural Eng..

[25] Ding, Y. *et al.* TSception: A deep learning framework for emotion detection using EEG. In 2020 international joint conference on neural networks (IJCNN), 1–7, 10.1109/IJCNN48605.2020.9206750 (2020).

[26] Palazzo S (2021). Decoding brain representations by multimodal learning of neural activity and visual features. IEEE Trans. Pattern Anal. Mach. Intell..

[27] Song Y, Zheng Q, Liu B, Gao X (2023). EEG conformer: Convolutional transformer for EEG decoding and visualization. IEEE Trans. Neural Syst. Rehabil. Eng..

[28] Mishra, A., Raj, N. & Bajwa, G. Eeg-based image feature extraction for visual classification using deep learning (2022). arXiv: 2209.13090.

[29] Chollet, F. Xception: Deep learning with depthwise separable convolutions (2017). arXiv: 1610.02357.

[30] Szegedy, C. *et al.* Going deeper with convolutions. In 2015 IEEE conference on computer vision and pattern recognition (CVPR), 1–9, 10.1109/CVPR.2015.7298594 (2015).

[31] Schirrmeister RT (2017). Deep learning with convolutional neural networks for eeg decoding and visualization. Human Brain Map..

[32] Zhang H, Silva FHS, Ohata EF, Medeiros AG, Rebouças Filho PP (2020). Bi-dimensional approach based on transfer learning for alcoholism pre-disposition classification via eeg signals. Front. Human Neurosci..

[33] Kingma, D. P. & Ba, J A method for stochastic optimization, Adam, 2017), arXiv: 1412.6980.

[34] Ng, A. Y. Feature selection, l1 vs. l2 regularization, and rotational invariance. In proceedings of the twenty-first international conference on machine learning, ICML ’04, 78, 10.1145/1015330.1015435 (Association for computing machinery, New York, NY, USA, 2004).

[35] Smith, L. N. & Topin, N. Super-convergence: Very fast training of neural networks using large learning rates (2018). arXiv: 1708.07120.

[36] Rombach, R., Blattmann, A., Lorenz, D., Esser, P. & Ommer, B. High-resolution image synthesis with latent diffusion models. In 2022 IEEE/CVF conference on computer vision and pattern recognition (CVPR)

[37] Ho, J., Jain, A. & Abbeel, P. Denoising diffusion probabilistic models. In Proceedings of the 34th international conference on neural information processing systems

[38] Ronneberger O, Fischer P, Brox T, Navab N, Hornegger J, Wells WM, Frangi AF (2015). U-net: Convolutional networks for biomedical image segmentation. Medical image computing and computer-assisted intervention - MICCAI 2015.

[39] Esser, P., Rombach, R. & Ommer, B. Taming transformers for high-resolution image synthesis. In 2021 IEEE/CVF conference on computer vision and pattern recognition (CVPR), 12868–12878 (IEEE, New York, 2021).

[40] Dhariwal, P. & Nichol, A. Diffusion Models Beat GANs on Image Synthesis. In Ranzato, M., Beygelzimer, A., Dauphin, Y., Liang, P. S. & Vaughan, J. W. (eds.) Advances in Neural Information Processing Systems, 8780–8794 (Curran Associates, Inc., 2021).

[41] Liu, L., Ren, Y., Lin, Z. & Zhao, Z. Pseudo numerical methods for diffusion models on manifolds (2022). arXiv: 2202.09778.

[42] Dosovitskiy, A. *et al.* An image is worth 16x16 words: Transformers for image recognition at scale (2021). arXiv: 2010.11929.

[43] Ahmed, H., Wilbur, R. B., Bharadwaj, H. M. & Siskind, J. M. Object classification from randomized eeg trials. In 2021 IEEE/cvf conference on computer vision and pattern recognition (CVPR), 3844–3853, 10.1109/CVPR46437.2021.00384 (2021).

[44] Pontifex MB, Coffman CA (2023). Validation of the gtec unicorn hybrid black wireless EEG system. Psychophysiology.

[45] Zheng X, Chen W (2021). An attention-based bi-lstm method for visual object classification via eeg. Biomed. Signal Process. Control.

[46] Bai, Y. *et al.* Dreamdiffusion: Generating high-quality images from brain eeg signals (2023). arXiv: 2306.16934.

[47] Huang G (2023). Discrepancy between inter- and intra-subject variability in eeg-based motor imagery brain-computer interface: Evidence from multiple perspectives. Front. Neurosci..

[48] Petroni A (2018). The variability of neural responses to naturalistic videos change with age and sex. eNeuro.

[49] Smit DJA, Boomsma DI, Schnack HG, Hulshoff Pol HE, de Geus EJC (2012). Individual differences in eeg spectral power reflect genetic variance in gray and white matter volumes. Twin Res. Human Genet..

[50] Kriegeskorte N (2008). Matching categorical object representations in inferior temporal cortex of man and monkey. Neuron.

[51] Jozwik KM (2022). Disentangling five dimensions of animacy in human brain and behaviour. Nat. Commun. Biol..

[52] Bird CM, Berens SC, Horner AJ, Franklin A (2014). Categorical encoding of color in the brain. Proc. Natl. Acad. Sci..

[53] Pasupathy A, Kim T, Popovkina DV (2019). Object shape and surface properties are jointly encoded in mid-level ventral visual cortex. Curr. Opin. Neurobiol..

[54] Roe AW (2012). Toward a unified theory of visual area v4. Neuron.

[55] Ozcelik, F., Choksi, B., Mozafari, M., Reddy, L. & VanRullen, R. Reconstruction of perceived images from fmri patterns and semantic brain exploration using instance-conditioned gans. In 2022 international joint conference on neural networks (IJCNN), 1–8, 10.1109/IJCNN55064.2022.9892673 (2022).

[56] Teichmann L (2020). The influence of object-color knowledge on emerging object representations in the brain. J. Neurosci..

[57] Contini EW, Wardle SG, Carlson TA (2017). Decoding the time-course of object recognition in the human brain: From visual features to categorical decisions. Neuropsychologia.

[58] Malach R, Levy I, Hasson U (2002). The topography of high-order human object areas. Trends Cogn. Sci..

[59] Sauer, A., Lorenz, D., Blattmann, A. & Rombach, R. Adversarial diffusion distillation (2023). arXiv: 2311.17042.

[60] Kavasidis, I., Palazzo, S., Spampinato, C., Giordano, D. & Shah, M. Brain2image: Converting brain signals into images. In proceedings of the 25th ACM international conference on multimedia, MM ’17, 1809-1817, 10.1145/3123266.3127907 (Association for computing machinery, New York, NY, USA, 2017).

[61] Cui, W. *et al.* Neuro-gpt: Developing a foundation model for eeg (2023). arXiv: 2311.03764.

