# Supplementary material for: Image classification and reconstruction from low-density EEG
Source: Sci Rep. 2024 Jul 16;14:16436. doi: 10.1038/s41598-024-66228-1 (PMC11252274; doi:10.1038/s41598-024-66228-1)
Supplement: Supplementary file 1 — Supplementary Information. [file 41598_2024_66228_MOESM1_ESM.pdf]

Image Classification and Reconstruction from Low-Density EEG

Sven Guenther[1,*], Nataliya Kosmyna[2,], and Pattie Maes[2]**

[1]School of Computation, Information and Technology, Technical University of Munich, Germany

[2]Media Lab, Massachusetts Institute of Technology, USA

* sven.guenther@tum.de
** nkosmyna@mit.edu

Supplementary Information

Image Selection: Similarity score to select samples for each image class

To compute the similarity score, we first extracted a lower dimensional feature vector for each image, by passing it through the ResNet50 model[1]. The ResNet50 model was pretrained to classify ImageNet instances, however, we discarded the final Softmax layer, to obtain the vector output. Subsequently, we computed the cosine similarity, displayed in Equation (1), between the prototype image vector and all other image vectors of the same category and gathered the 49 most similar images. In this way, we were able to exclude copies of the same image and could hand-select the 30 most coherent and representable samples out of the 50 images per class.

$$\cos(\theta) = \frac{A \cdot B}{\|A\|\|B\|} = \frac{\sum_{i=1}^{n} A_i B_i}{\sqrt{\sum_{i=1}^{n} A_i^2}\sqrt{\sum_{i=1}^{n} B_i^2}} \qquad (1)$$

Preprocessing: Filtering

We applied a FIR highpass and lowpass filter with a Kaiser window and cutoff-frequencies of 1Hz and 95Hz, respectively. The high- and lowpass filters were employed separately, as the highpass filter required a stronger and faster attenuation than the lowpass filter to attenuate low frequencies. In contrast, the lowpass filter could have a shallower transition that introduces less smearing of the signal[2]. Therefore, the lowpass filter had a transition width of 6 Hz, compared to a 2 Hz transition width for the high pass filter. To filter out line noise, we utilized an IIR notch filter at 60Hz. To maintain the signal shapes and feature locations, we employed zero-phase filtering. For the filter implementation, we utilized the SciPy Python package[3]. The frequency and time responses of the filters, can be seen in Supplementary Fig. 2. Notably, filtering was employed before splitting the recording into trials to avoid edge artifacts[4].

Preprocessing: Bad trial rejection

For the bad trial rejection, we first filtered the data as we aimed to assess the trials solely based on their SNR in the frequency regions of interest. Then, we segmented the signals into trials and discarded the parts recorded before and after the experiment, as well as the sections overlapping with the gray screen displays in between images. Each trial was successively checked for non-numeric (NaN) and flat values. A channel was considered to be flat if either the median absolute deviation from the median or the standard deviation of the samples was below a predefined threshold (1e-9 microvolts) for one of the channels. Whereas NaN values indicated connection problems between the EEG and the experiment computer, flat channels implied a loss of contact between an electrode and the scalp. Both trial rejection methods were adapted from an existing preprocessing pipeline[5]. Additionally, we applied a trial rejection based on correlations between the electrodes. EEG signals measured from the scalp commonly show a high correlation, especially if they are located in close proximity[6]. Therefore, we checked if the electrode correlations in a trial were in a reasonable range. If one or more of the channels would show a low absolute correlation (<0.3) with the other channels, this could indicate a loss of contact with the scalp. On the other hand, if a channel perfectly correlates with another electrode, this might signify unwanted connections of channels. The latter case could be due to the conductive gel creating a connection between multiple channels. The choice of the upper correlation bound differed based on whether the recordings were done with or without gel and varied per subject. We plotted the rejected trials for different bounds for every participant and decided for each bound whether the rejection was reasonable by visual inspection. All chosen upper bounds were in the range of 0.95 to 0.98. The output of the bad trial rejection was a mask for each recording that marked channels as bad for later exclusion.

Classification: Model-specific adaptations

Here we present the model-specific modifications that ensured the seamless integration with our framework and allowed for exploration of optimal hyperparameter combinations. As a general adaptation, we changed the channel-input size to work with our 8-electrode setup.

EEGNet

For the hyperparameter search, we varied the number of temporal filters (F_1) in the first convolutional block, but kept the number of spatial filters ($D = 2$) per temporal filter constant, similar to Lawhern et al.[7]. However, we explored a larger search space for the number of temporal filters compared to the original study to explore more complex architectures. The number of pointwise convolutions in the second convolutional block was set to $F_2 = F_1 * D$, as proposed by Lawhern et al.[7], who used the same notation. Moreover, we experimented with different Dropout probabilities to study the impact of regularization on the model's ability to generalize. For that, we adopted the probability values that the authors used for cross-subject (0.25) and within-subject classifications (0.5) as the search space. Additionally, we alternated between average and max pooling to gauge the effect of selecting the most prominent feature in the intermediate inputs or an average of the features. The other hyperparameters, like kernel dimensions for the convolutional and pooling layers, were adopted from Lawhern et al.[7].

TSCeption

For TSCeption, we experimented with the depth of the temporal (T) and spatial (S) convolutions to alternate the model's complexity. We further varied the ratio coefficients that determined the different kernel dimensions of the temporal convolutions to alter the temporal patterns that could be extracted. Ding et al.[8] have only employed ratio coefficients of $\alpha_r = [0.5, 0.25, 0.125]$ with regards to the sampling rate, to capture frequency at 2 Hz to above, 4 Hz to above, and 8 Hz to above, respectively. As previous studies have emphasized the relevance of gamma frequencies for visual classification[9], we wanted to explore distinct filters for higher frequencies, as well. In contrast to the approach by Ding et al.[8], which divided the local spatial filters by hemisphere, we applied them separately to the four inferiorly located and the four superiorly located EEG channels over the occipital and parieto-occipital lobe. The strategy was to isolate activity originating from the primary visual cortex through inferiorly located electrodes and to discern activity from advanced visual areas using more superiorly located channels. Furthermore, we explored different Dropout probabilities to gauge the regularization effect on the model performance.

EEG-ChannelNet

Compared to the original implementation of EEG-ChannelNet by Palazzo et al.[10], we varied the number (n) and depth (d) of the convolutions in the temporal (T) and spatial block (S). With regards to the spatial block, we had to reduce the number of filters, as the model was developed to work with a spatial dimension of 128 and applied four spatial convolution operations with kernel sizes of (128, 1), (64, 1), (32, 1) and (16, 1), respectively. For our setup with only 8 electrodes, we adapted the number of spatial filters as well as the kernel sizes. Thus, we either used two spatial convolutions of (8, 1) and (4, 1), or we added a third with a kernel dimension of (2, 1). Additionally, we adapted the padding employed for the spatial convolutions to (4, 0), (2, 0), and (1, 0) to fit with the aforementioned spatial kernels. Furthermore, to limit the computational complexity of the model, we reduced the number of residual blocks from 4 to 2 and modified the classifier head from Palazzo et al.[10]. The initial classifier head included a hidden layer, which we changed to directly map to the output. Similar to the previous models, we experimented with different Dropout probabilities.

EEG Conformer

In its original form, the depth of the convolutional operations and the embedding size of the EEG Conformer have been set to the same value ($k = 40$)[11]. We decided to study the effect of the convolution module depth and the embedding size, separately. Therefore, we varied the depth of the temporal and spatial convolution (k_{conv}) and employed the 1x1 convolution to map to a differing embedding size (k_{emb}). Additionally, the spatial kernel size was changed from (22, 1) to (8, 1) to fit with our channel count. As Song et al.[11] have shown the insensitivity of the Conformer to the self-attention depth and number of heads on two different EEG datasets, we adopted their settings of the attention block. However, we varied the pooling kernel size of the preceding convolutional block, which determined one of the dimensions of the input for the attention process. In their study, Song et al.[11] noted that the variation of the kernel sizes (m) between 15 to 45, in increments of 10, significantly affected the classification accuracy. Therefore, we defined the search space for the pooling kernel in that range. Notably, for the EEG Conformer, we did not use 30 epochs as for the other models, as the transformer requires more training time than purely CNN-based models[11]. Instead, we checked for super convergence using 50 epochs, but also allowed 200 epochs. The latter value was derived from the original paper showing convergence between 200 to 250 epochs. All other model-specific hyperparameters were adopted from Song et al.[11].

EEG-to-Image-based model

Different from the previous approaches, the EEG-to-Image-based algorithm consisted of a pre-trained deep learning model employed for feature extraction and a machine learning classifier for the actual classification. Due to the different input dimensions, we had to adapt the EEG-to-Image transformation process. Mishra et al.[12] vertically stacked four copies of the transformed channel data and then vertically combined the stacked copies from each channel before resizing to (224, 224, 3). Instead, we vertically packed 28 copies per channel, mapping to a dimension of 224, and then resized the temporal dimension from 500 to 224, to obtain a similar outcome with fewer input channels.

Mishra et al.[12] have reported the best performance using the pre-trained EfficientNet model[13] for feature extraction and an SVM as the classifier. Therefore, we only adopted that approach from the original study. However, we additionally did a hyperparameter search for the machine learning classifier. The SVM was tested with different kernels and various values for the C regularization parameter and the gamma kernel coefficient. The C and gamma hyperparameters were sampled from log-uniform distribution-returning values between e^{-10} to e^{10}, as previous researchers have recommended using exponentially growing search spaces[14]. The hyperparameter names were adopted from the Scikit-Learn framework[15].

Hyperparameter Search

To test different hyperparameter combinations for each of the five models, we implemented a random search using Weights and Biases. The search space for each model can be found in Supplementary Table 2.

For each model, we first conducted 100 runs with randomly sampled hyperparameters from the predefined search space. Then, we inspected the validation loss for the respective combinations to refine the search space, before carrying out additional 50 runs to find the best hyperparameter set. As the optimal hyperparameter combination may differ depending on the dataset, we conducted the hyperparameter search for each subject individually. We did not have specific expectations on how the models with their respective hyperparameter combinations would perform since we tried them on a completely new dataset. Additionally, except for the EEGNet, most models either have only been tested on a flawed dataset (EEG-ChannelNet and EEG-to-Image) or have been used in non-stationary EEG tasks (TSCeption and Conformer).

Why the EEG-to-Image framework might be at chance level prediction

The EEG-to-Image classification paradigm remained around chance level performance in our analysis. As discussed before, the high accuracy reported in Mishra et al.[12] has likely arisen from predicting based on block-level temporal correlations arising from the EEG hardware. Additionally, the pronounced difference between our EEG-to-Image classification performance compared to the results in Mishra et al.[12] might partly be due to the different hardware setup and especially the lower channel count. As we mentioned, due to the fewer electrodes, we had to increase the number of times each channel was stacked vertically. However, we further question the effectiveness of the method for the following reason. As the authors have not specified which layer of the pre-trained image model, they have used to extract features from the transformed EEG images, we assumed it would be the pre-classification layer. This assumption was reasonable as this layer had the smallest dimension of all layers (1280) and no additional dimensionality reduction methods were mentioned to mitigate the curse of dimensionality. However, in this layer, the pre-trained network already extracted high-level information to classify a real image as belonging to a certain category of the ImageNet dataset. Notably, the 8-bit-grayscale transformation of the EEG yielded a spectrogram-looking output that was more abstract than images in ImageNet. In terms of assigning a category to the abstract input, we consider it to be unlikely that the small nuances in the transformed EEG images would yield drastically different output node activations. Therefore, the SVM received non-informative feature vectors leading to random predictions. Potentially, selecting an earlier layer from the pre-trained model that would extract low-level features and applying a dimensionality reduction technique before feeding the output to an SVM could be more successful.

Supplementary Figures

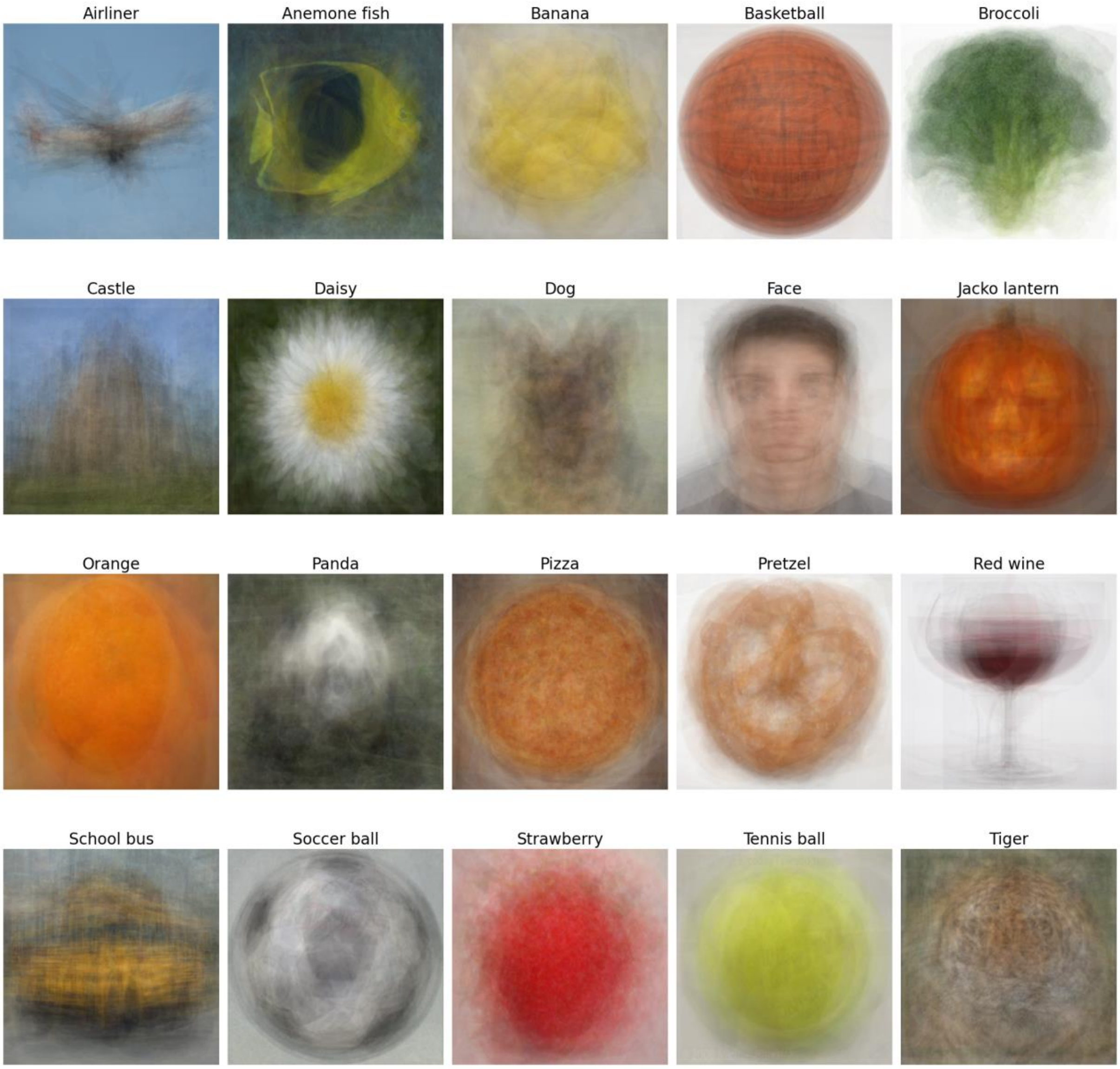

Supplementary Figure 1. Averaged image of all images of the respective object categories

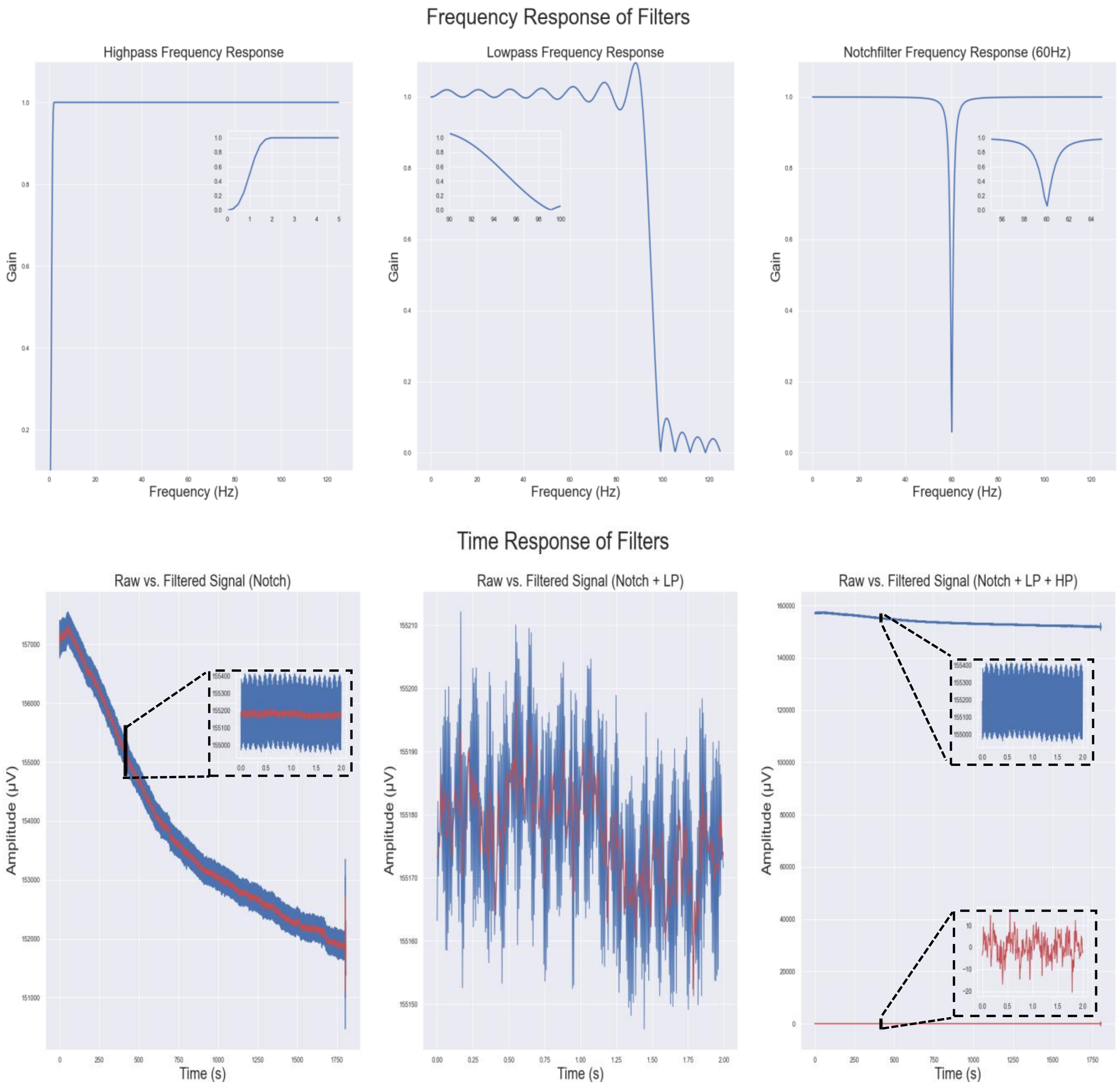

Supplementary Figure 2. Frequency and time response of the employed filters. Small graphs inside each figure depict a zoomed-in version. The time responses display the notch-filtered signal in red overlayed on the raw signal in blue, the notch and low passed filtered signal over the raw signal, and the notch, lowpass, and high passed signal next to the raw signal, respectively, from left to right. We used a second-order IIR notch filter at 60 Hz, as well as two separate FIR highpass and lowpass filters with a Kaiser window and a cutoff frequency at 1 Hz and 95 Hz, respectively. The filters were applied forward and backward with the filtfilt function from SciPy, such that the combined filter had a net zero-phase effect.

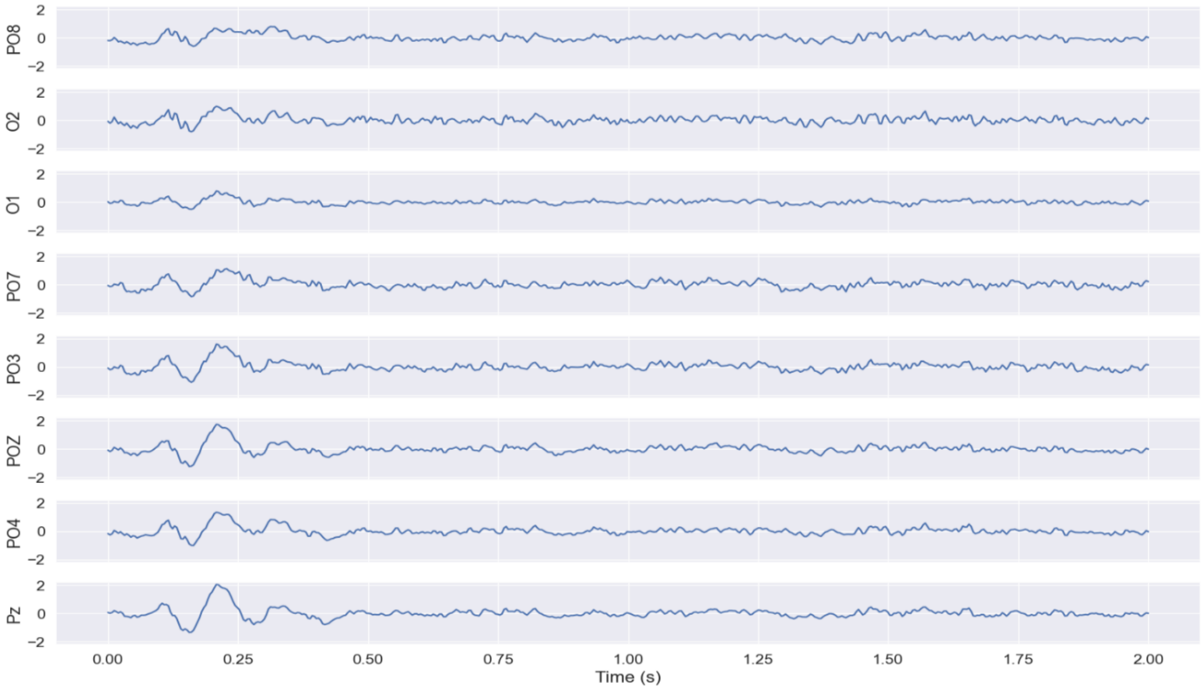

Supplementary Figure 3. Mean preprocessed EEG signal per channel over first image category ("airliner") in a wet recording from Subject 1. The y-axis specifies the electrode positions and contains the mean normalized amplitude, while the x-axis shows the time in seconds.

A clear event-related potential is visible between ca. 100 to 300 ms with a distinct negative trough at about 100 ms and a positive peak around 200 ms. Both the timing (Kaneshiro et al., 2015; Simanova et al., 2010), as well as the typical N1-P2 onset response (Ahmed et al., 2021) are in line with previous research. Both findings suggest that the recorded data successfully captured activity evoked by the presented images.

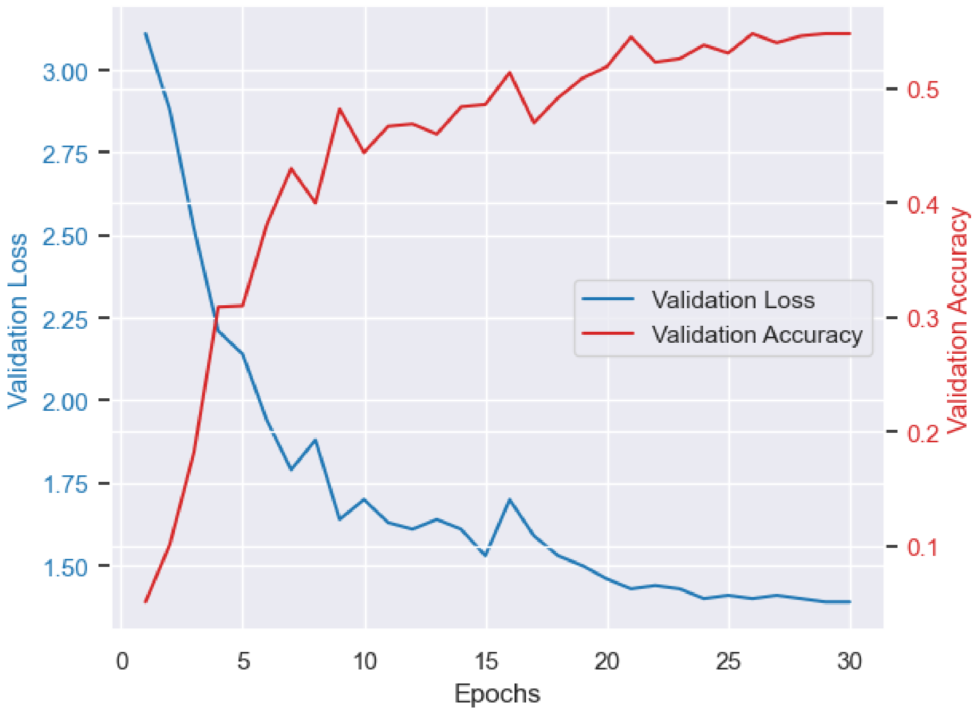

Supplementary Figure 4. Validation loss and accuracy during training of EEGNet on wet recordings of Subject 1

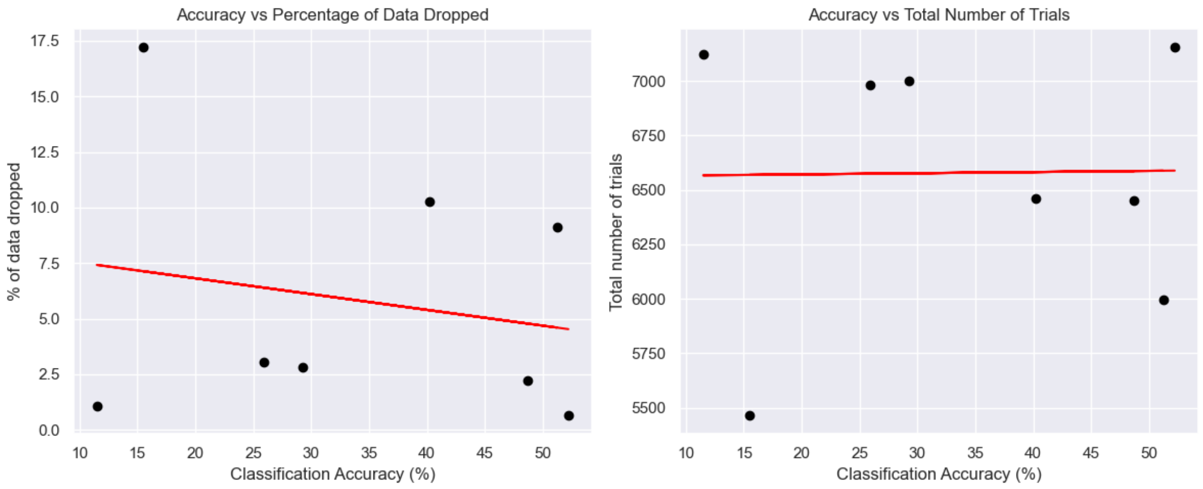

Supplementary Figure 5. Scatter plots showing the relation between the classification accuracy and the percentage of data dropped, as well as between the classification accuracy and the total number of trials. Each dot corresponds to one subject. The red line was fitted to the data to visualize the overall trend. The Spearman rank-order correlation between the accuracy and percentage of dropped data and between the accuracy and the total number of trials were non-significant with r=-0.24, p=0.57 and r=0.05, p=0.91, respectively. This result has to be treated with caution given the small sample size.

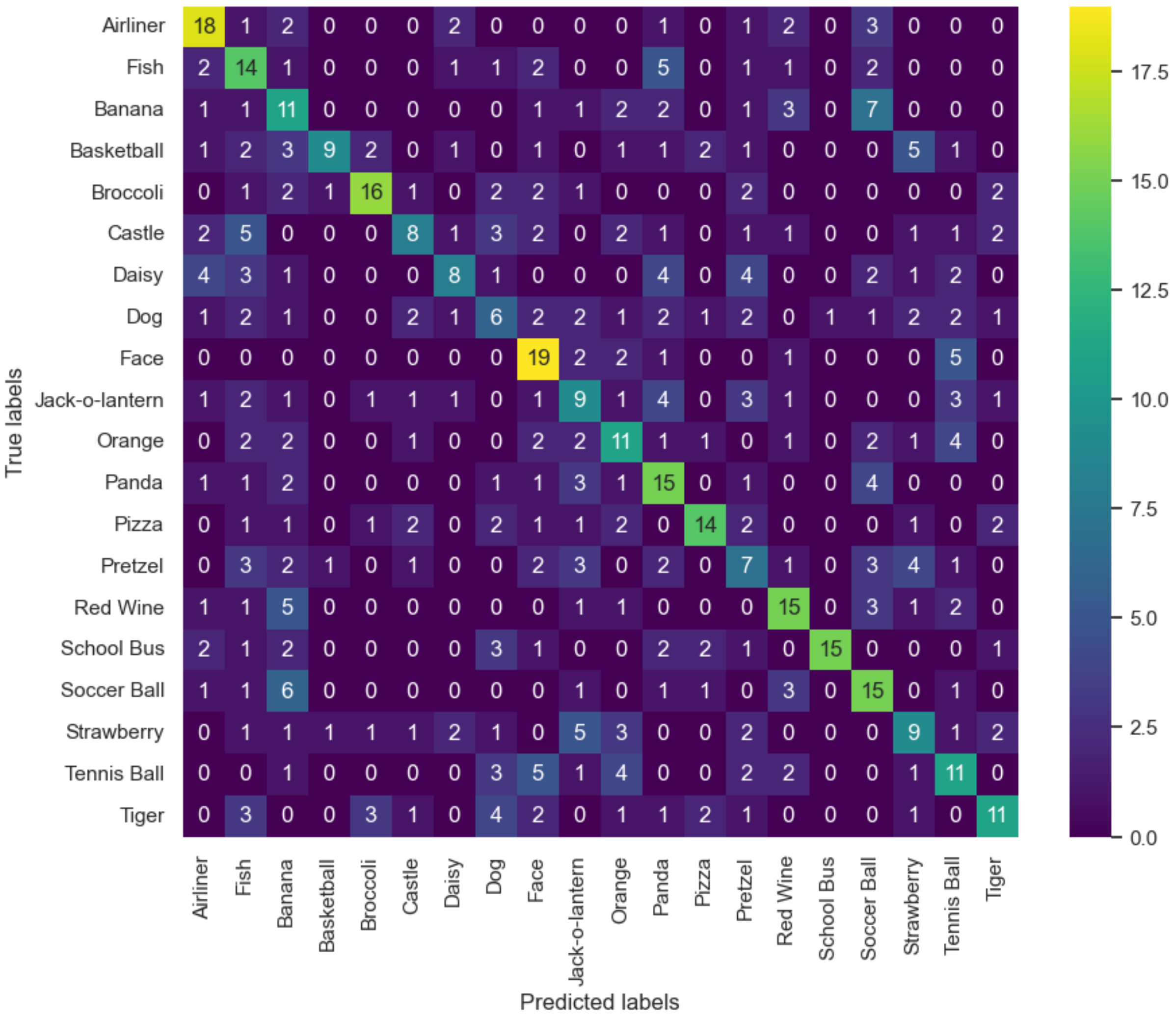

Supplementary Figure 6. Confusion matrix displaying true and predicted labels for each image class for the EEGNet model on the test recording from **Subject 2**. Ground truth labels are presented on the y-axis and predicted labels on the x-axis

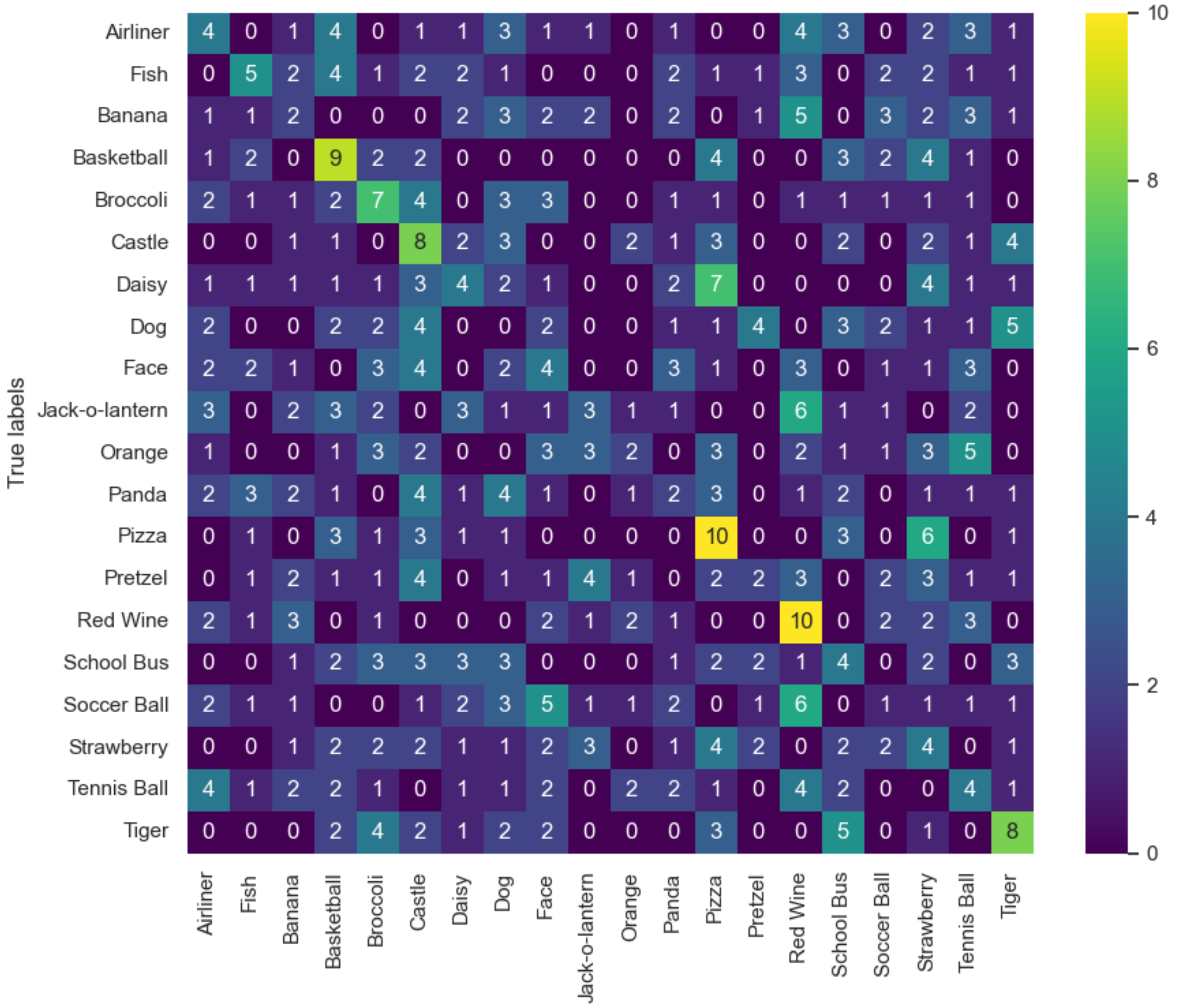

Supplementary Figure 7. Confusion matrix displaying true and predicted labels for each image class for the EEGNet model on the test recording from **Subject 3**. Ground truth labels are presented on the y-axis and predicted labels on the x-axis

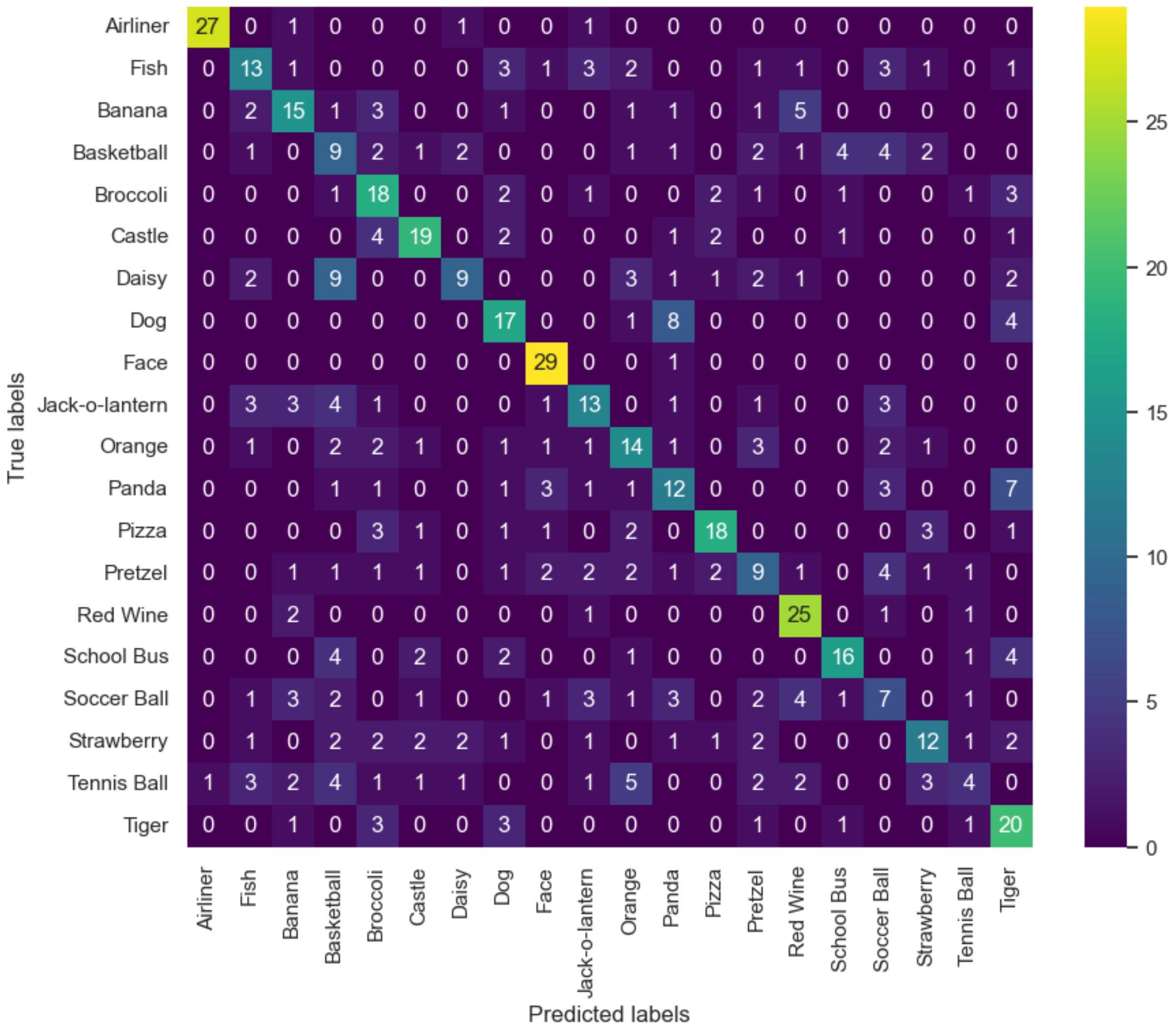

Supplementary Figure 8. Confusion matrix displaying true and predicted labels for each image class for the EEGNet model on the test recording from **Subject 4**. Ground truth labels are presented on the y-axis and predicted labels on the x-axis

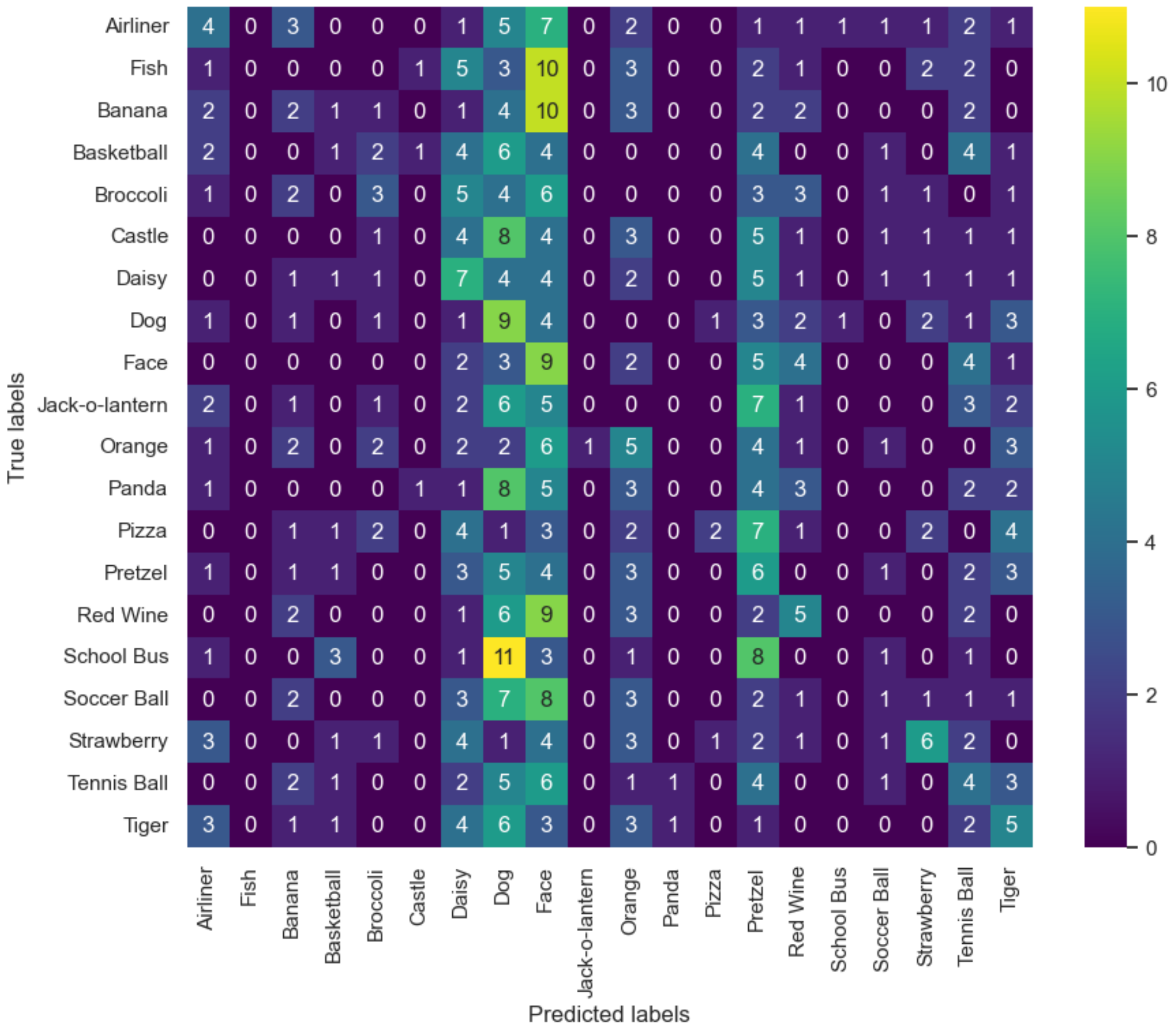

Supplementary Figure 9. Confusion matrix displaying true and predicted labels for each image class for the EEGNet model on the test recording from **Subject 5**. Ground truth labels are presented on the y-axis and predicted labels on the x-axis

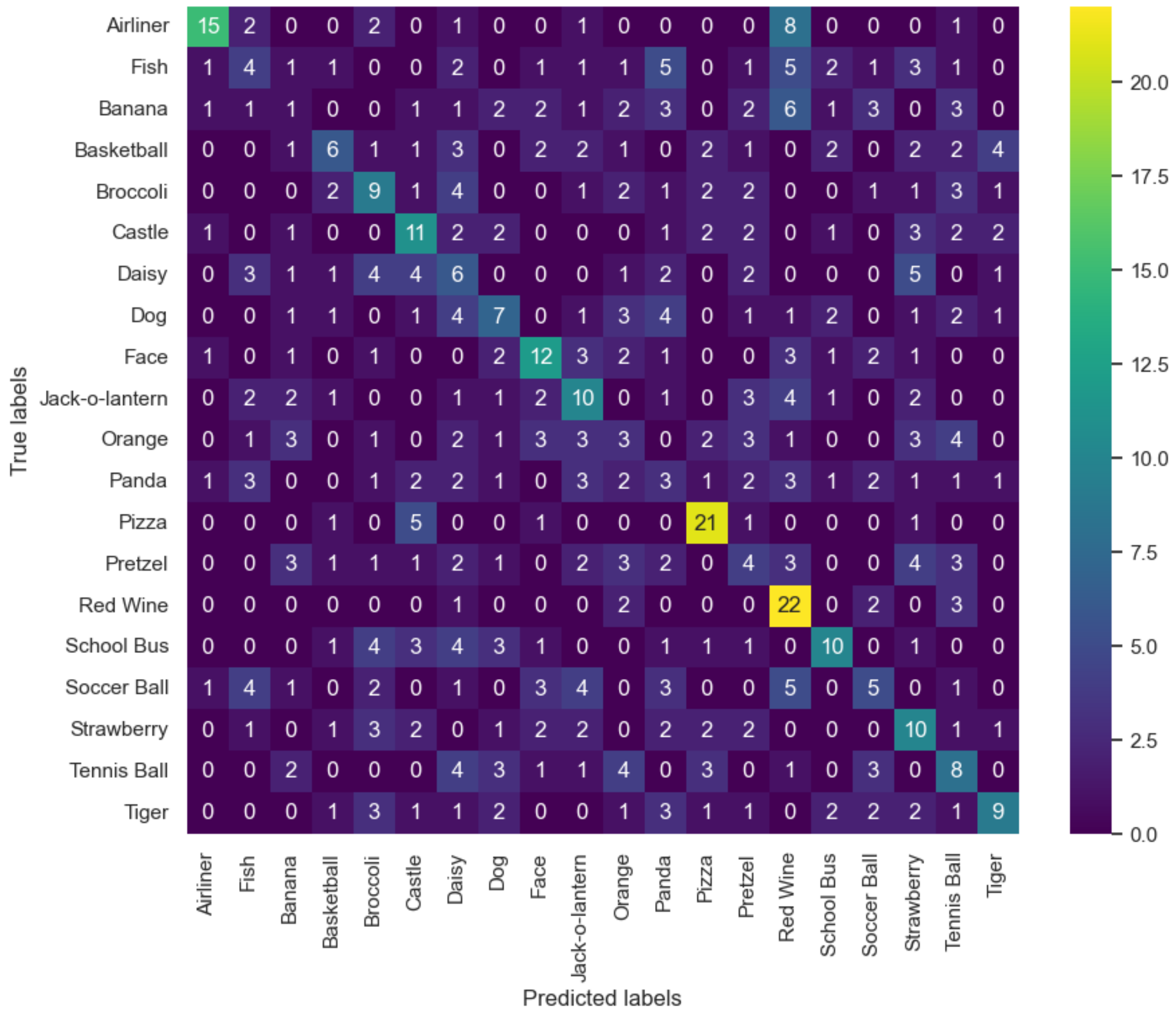

Supplementary Figure 10. Confusion matrix displaying true and predicted labels for each image class for the EEGNet model on the test recording from **Subject 6**. Ground truth labels are presented on the y-axis and predicted labels on the x-axis

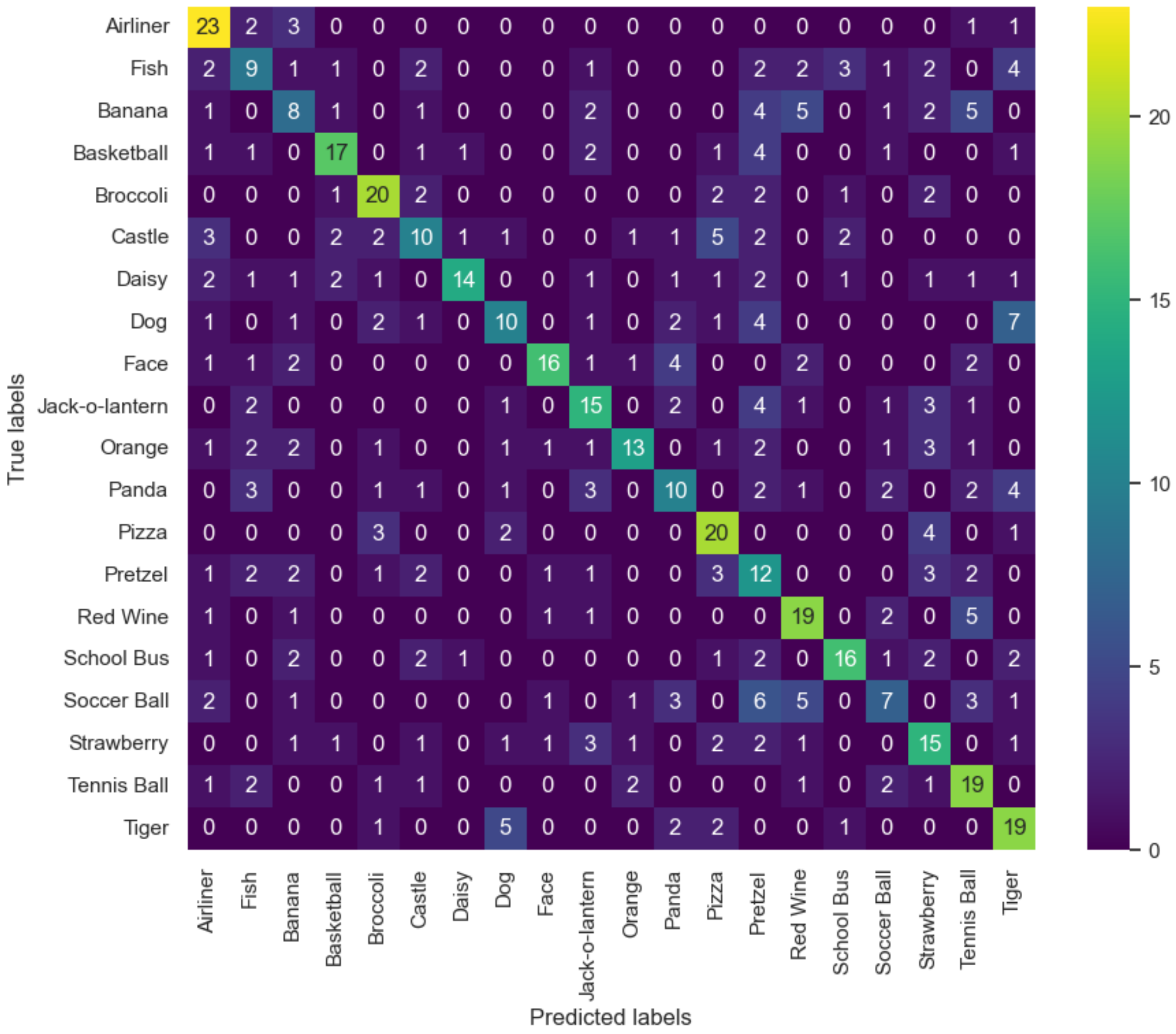

Supplementary Figure 11. Confusion matrix displaying true and predicted labels for each image class for the EEGNet model on the test recording from **Subject 7**. Ground truth labels are presented on the y-axis and predicted labels on the x-axis

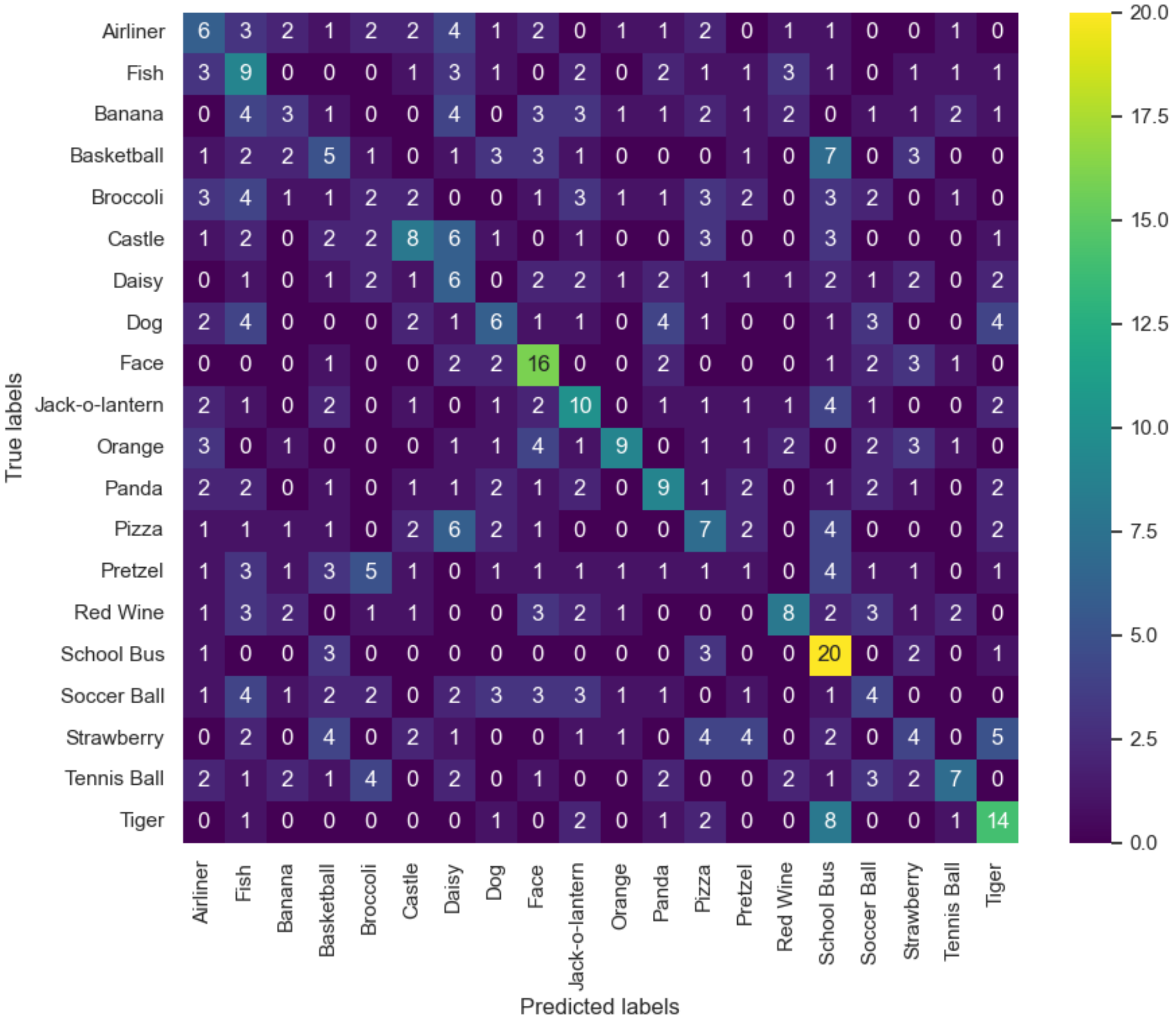

Supplementary Figure 12. Confusion matrix displaying true and predicted labels for each image class for the EEGNet model on the test recording from **Subject 8**. Ground truth labels are presented on the y-axis and predicted labels on the x-axis

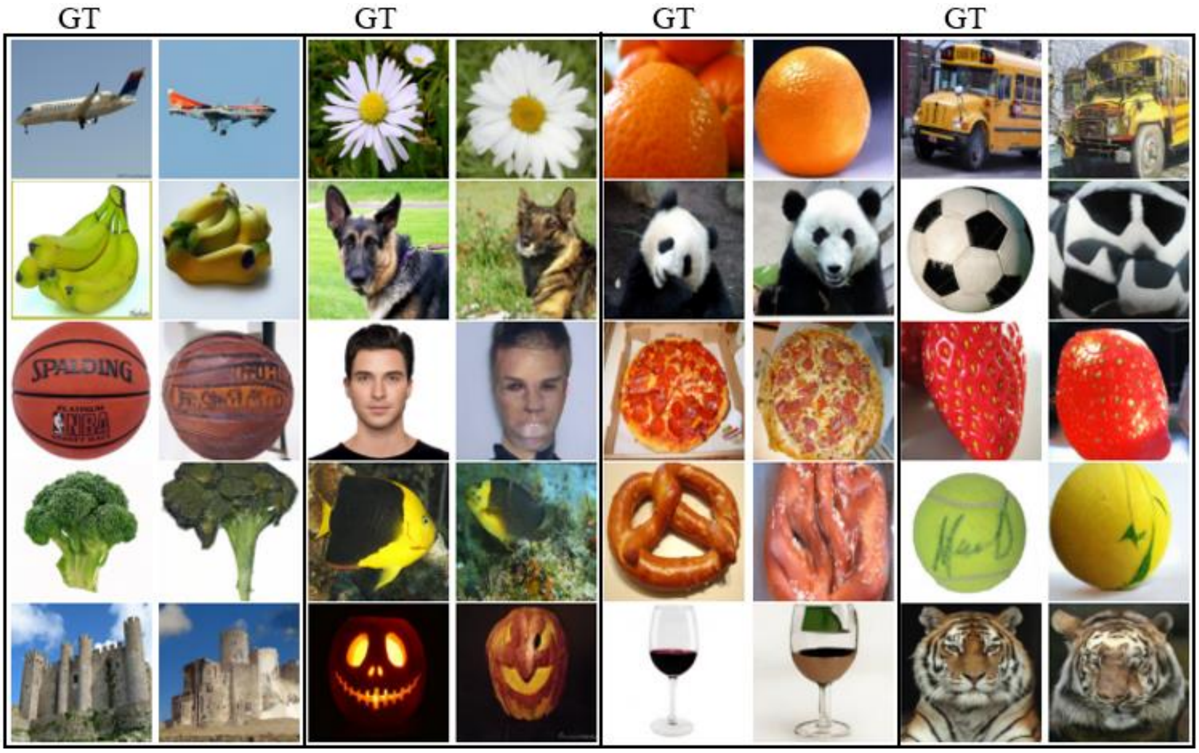

Supplementary Figure 13. Selection of good reconstructions from **Subject 7**, which had a 1000 trial 50-class top-1 accuracy on the best generated images of 30.3%, as well as an average 1000 trial 50-class top-1 accuracy across the five generated samples per image of 26.6%. The figure displays an example of each image class from the base test set. Ground truth (GT) is presented on the left with the reconstructed image to the right for each column, respectively. The presented ground truth images, except for the human face image, were taken from the ImageNet Large Scale Visual Recognition Challenge[16] dataset which permits usage for non-commercial research. The human face image was taken from the Human Faces dataset[17] published under CC0 license.

Supplementary Tables

Subject	% of data dropped	Total number of trials
P001	0.65	7153
P002	10.26	6461
P003	17.2	5465
P004	9.14	5997
P005	1.06	7124
P006	2.81	6998
P007	2.24	6452
P008	3.06	6980

Supplementary Table 1. Percentage of dropped trials of used recordings and total number of trials from each subject.

Note. Every subject underwent 12 recording sessions each containing 600 trials, amounting to a possible total trial number of 7200. For P003, P004 and P007 we had to exclude one recording due to a malfunctioning electrode. The percentage of dropped data is calculated with respect to the remaining 11 sessions for these participants to obtain an estimate of the quality of the utilized recordings.

Hyperparameter	Search Space
**EEGNet**	
F_1	[4, 8, 16, 32, 64]
Pooling	[max, average]
Dropout prob.	[0.25, 0.5]
**TSCeption**	
T	[3, 6, 9]
S	[9, 12, 15]
α_r	$\left[[0.5, 0.25, 0.125], [0.5, 0.25, 0.0625], [0.5, 0.25, 0.03125]\right]$
Dropout prob.	[0.2, 0.4, 0.6]
**EEG-ChannelNet**	
n_T	[2, 4, 6]
n_S	[2, 3]
d_T	[5, 10, 20]
d_S	[4, 8, 16]
Dropout prob.	[0.2, 0.4, 0.6]
**EEG Conformer**	
k_{conv}	[30, 35, 40, 45, 50]
k_{emb}	[30, 35, 40, 45, 50]
m	[15, 25, 35, 45]
epochs	[50, 200]
**EEG-to-Image**	
SVM_{kernel}	[linear, radial basis function]
SVM_C	x~Log-Uniform(e^{-10}, e^{10})
SVM_{gamma}	x~Log-Uniform(e^{-10}, e^{10})

Supplementary Table 2. Hyperparameter search space for the classification models

References

1. He, K., Zhang, X., Ren, S., & Sun, J. Deep residual learning for image recognition. *2016 IEEE Conference on Computer Vision and Pattern Recognition (CVPR)*, 770–778, DOI: https://doi.org/10.1109/CVPR.2016.90 (2016).

2. Widmann, A., Schröger, E., & Maess, B. Digital filter design for electrophysiological data - a practical approach. *J. Neuroscience Methods*, **250**, 34–46, DOI: https://doi.org/10.1016/j.jneumeth.2014.08.002 (2015).

3. Virtanen, P., et al. (2020). SciPy 1.0: Fundamental algorithms for scientific computing in Python. *Nat. Methods,* **17**, 261–272. https://doi.org/10.1038/s41592-019-0686-2

4. van Driel, J., Olivers, C. N. L., & Fahrenfort, J. J. High-pass filtering artifacts in multivariate classification of neural time series data. *J. Neuroscience Methods*, ***352***, DOI: https://doi.org/10.1016/j.jneumeth.2021.109080 (2021).

5. Bigdely-Shamlo, N., Mullen, T., Kothe, C., Su, K.-M., & Robbins, K. A. The PREP pipeline: standardized preprocessing for large-scale eeg analysis. *Front. Neuroinformatics,* **9**, DOI: 10.3389/fninf.2015.00016 (2015).

6. Brunner, C., Billinger, M., Seeber, M., Mullen, T. R., & Makeig, S. Volume conduction influences scalp-based connectivity estimates. *Front.Computational Neuroscience*, **10**, DOI: https://doi.org/10.3389/fncom.2016.00121 (2016).

7. Lawhern, V. J., Solon, A. J., Waytowich, N. R., Gordon, S. M., Hung, C. P., & Lance, B. J. EEGNet: A compact convolutional neural network for EEG-based brain–computer interfaces. *J. Neural Engineering*, ***15***, DOI: https://doi.org/10.1088/1741-2552/aace8c (2018).

8. Ding, Y., Robinson, N., Zeng, Q., Chen, D., Wai, A. A. P., Lee, T.-S., & Guan, C. TSception: A deep learning framework for emotion detection using EEG. *2020 International Joint Conference on Neural Networks (IJCNN)*, 1-7, DOI: https://doi.org/10.1109/IJCNN48605.2020.9206750 (2020).

9. Muthukumaraswamy, S. D., & Singh, K. D. Visual gamma oscillations: the effects of stimulus type, visual field coverage and stimulus motion on MEG and EEG recordings. *NeuroImage*, **69**, 223–230, DOI: https://doi.org/10.1016/j.neuroimage.2012.12.038 (2013).

10. Palazzo, S., Spampinato, C., Kavasidis, I., Giordano, D., Schmidt, J., & Shah, M. Decoding brain representations by multimodal learning of neural activity and visual features. *IEEE Transactions on Pattern Analysis & Machine Intelligence*, **43**, 3833-3849, DOI: https://doi.org/10.1109/TPAMI.2020.2995909 (2021).

11. Song, Y., Zheng, Q., Liu, B., & Gao, X. EEG conformer: Convolutional transformer for EEG decoding and visualization. *IEEE Transactions on Neural Systems and Rehabilitation Engineering*, **31**, 710-719, DOI: https://doi.org/10.1109/TNSRE.2022.3230250 (2023).

12. Mishra, A., Raj, N., & Bajwa, G. EEG-based image feature extraction for visual classification using deep learning. Preprint at https://doi.org/10.48550/arXiv.2209.13090 (2022).

13. Tan, M., & Le, Q. V. EfficientNet: Rethinking model scaling for convolutional neural networks. *Proceedings of the 36th International Conference on Machine Learning, ICML 2019*, 6105-6114, http://proceedings.mlr.press/v97/tan19a.html (2019).

14. Hsu, C.-W., Chang, C.-C., & Lin, C.-J. *A practical guide to support vector classification*. Department of Computer Science, National Taiwan University, http://www.csie.ntu.edu.tw/~cjlin (2003).

15. Pedregosa, F., et al. Scikit-learn: Machine learning in Python. *J. Machine Learning Research*, **12**, 2825–2830, (2011).

16. Russakovsky, O., et al. Imagenet large scale visual recognition challenge. International Journal of Computer Vision, 115, 211-252. DOI: 10.1007/s11263-015-0816-y (2015).

17. Gupta, A., Human faces [dataset]. *Kaggle* (accessed January 10, 2024). https://www.kaggle.com/datasets/ashwingupta3012/human-faces.
